# Analytical Reviews of the More Important Works on Cholera

**Published:** 1832-01-01

**Authors:** 


[Mr. KENNEDY.]

I. We shall take these publications without respect to order, priority, or rank, and commence with that of Mr. Kennedy.* This gentleman's volume is chiefly a compilation from the Indian reports, mixed with a certain portion of the compiler's own opinions and reasonings. We wish we could give unqualified praise to both or either of these portions. The abstracts from the Indian reports on cholera are conducted on a very dangerous plan. There are no, or very few, inverted commas—and although he avers that he uses the words of the authors, yet he also informs us that he has abridged them *more than two-thirds*—that he has occasionally departed from the text—but not to "interfere with the sense of the passage." Mr. K. tells us that he is indebted to the Court of Directors, for the opportunity of consulting the documents compiled in India by their command.

"In making abstracts from *a part* of these valuable reports, my object was of a general character, namely, to introduce short descriptions of cholera, and a variety of opinions from various authors—under the impression that a more comprehensive knowledge would be conveyed in this way of the peculiarities of the disease, than could possibly be afforded in the uniformity which usually pervades the description of an individual."—*Preface*, ii.

Considering that Mr. Kennedy jumped at once to the conclusion that

* The History of the Contagious Cholera; with Facts explanatory of its Laws, &c. Octavo, pp. 291. October, 1831.

cholera was a contagious disease, and placed it so even in the *title page*, notwithstanding that the question is still *sub judice*, our attention was arrested by the word "*part*" of the Indian Reports; and, on turning to the list of reports analyzed or condensed by Mr. Kennedy, we found them to amount to 37 in number. Of these 37, selected by our author from such an immense mass and multitude of reports and reporters, 22 authors are given who are neutral, either stating no opinion at all, respecting contagion, or being undecided—13 contagionists are brought on the tapis—*two* anti-contagionists!! Now, in the preface to the work, Mr. Kennedy lauds Mr. Scott, the reporter of the Madras Medical Board, as "claiming peculiar respect," and as having "executed a very difficult task with judgment and *impartiality*." Let us hear what Mr. Scott, as the organ of the Madras Board, says on this point. Our readers may turn to volume III, of this series, page 136, for particulars. Speaking of contagion, Mr. Scott observes:—

"If this question could have been decided simply by the opinions of a majority of medical men, it would have been already set at rest *against the doctrine of contagion or infection*;" "for there are few subjects, perhaps, on which so little diversity of sentiment has existed."*

Yet Mr. Kennedy has arrayed *two* individuals, among the army of medical officers in the East, as maintaining the doctrine of *non-contagion*!! After this specimen of candour and impartiality on the part of an author who purports the compilation of a work fairly and honestly from materials collected in the field of observation and experience, Mr. K. must excuse us for dismissing his book without further notice. It is not, in our opinion, constructed in a way to advance science or benefit humanity.

[Dr. A. Neale.]

II. We next come to the work of Dr. Adam Neale.† This is really a very curious, learned, and, considering the *grave* subject on which it treats, a very *amusing* production. We mean to give some account of it in a separate article, and therefore shall only notice, in this place, that portion of it which bears on the all-engrossing object of enquiry—Cholera Morbus.

From letters which we have recently received from several parts of the Continent, we find that the doctrine of "intro-animate pathology,"—or in other words, the doctrine of epidemic diseases being the result of animalcula or insects floating in the air, is becoming very prevalent there. In

---

* Why did Mr. Kennedy select his abstracted reports from the first two or three years of the epidemic in India, when mens' minds were distracted by the wide range and fatality of the disease? Why does he not advert to the opinion of Mr. Scott, whom he lauds for impartiality and judgment, whose report was drawn up *three years* later than the latest of Mr. Kennedy's reports? How could Mr. K. have had access to the documents at the India-house, and yet neglect the valuable reports in Mr. Scott's work? Even this Journal (vol. III.) would have furnished him with an analysis of Mr. Scott's work, if he could not procure the original.

† Researches to Establish the Truth of the Linnæan Doctrine of Animate Contagions, &c. &c. &c. 8vo. pp. 258. October, 1831.

the absence of a better theory, we should not be surprised to find the said doctrine extending to this country — perhaps even before the epidemic reaches these shores.

It is necessary to premise, as indeed the title of the work indicates, that the theory here propounded, originated, or at least was first brought into notice by the immortal Linnæus in a thesis of one of his disciples (Nysander) in the year 1575. Of this thesis Dr. Neale has given us some account. The following extract will convey some idea of this animalcular doctrine.

"Contagious diseases then, for the most part, all coincide in this peculiarity, that they are attended with exanthematous eruptions, either externally or internally, insomuch that even hydrophobia is said to produce certain pustules underneath the tongue; so that in all contagious maladies there exists a certain exanthematous matter, the eruption of which usually mitigates the febrile symptoms;* that the presence of this matter excites bodily uneasiness, if not feverish actions, and that, even in its mildest form, the disease is exacerbated at some certain hour of the day, or towards evening. That the power of the disease is increased by *sweet* articles, but diminished by *bitters*, and that the irritation is excited by *fat* articles of diet. That it is somewhat repelled by cold, but like every thing animate, is cherished by warmth, although it appears to be expelled by great heat: thus, from the application of heat, the itch and every exanthema is augmented, and from increase of heat and fever driven outwards to the warm surface of the body. That they are destroyed by *Anthelmintic* remedies, and therefore *Sulphureous* remedies cure the itch: and thus *Mercurial* medicines, (as they are destructive to nearly all insects), cure both the itch and syphilis, and prove preservative both from the plague and small-pox. And that as tobacco kills all minute insects, fumigation with it prevents contagion. That both anology and the sense of itching in the pustules of contagious diseases, seem to warrant the same inference. That both in itch and dysentery, the existence of minute insects has been the subject of ocular demonstration. That Langius had observed them in measles; and Kircher in the plague; Hauptmann in syphilis (animalcules resembling slugs); Zeigler in pœtechiæ; Lusitanus and Porcellus in small-pox, and that the last mentioned had observed also worms in cases of serpigo and other cutaneous diseases.

Both from analogy therefore, as well as from all the experience hitherto acquired, we may very easily believe that very minute insects of that kind, such for instance as *Acari* of various species, may be the causes of several contagious diseases. Nor is their size or structure at all repugnant to such an idea—since there are animalcules so minute that the human eye unassisted by the microscope cannot even perceive them. The lynx-sighted Lewenhoek observed thousands of insects (or animalcules more properly speaking) which added together scarcely equalled in size the hundredth part of a grain of sand, and even in those fluids which to the naked eye seem altogether pure and diaphonous. While the accurate and ingenious Reaumur has stated it to be his opinion, that that obscure vapour which somewhat darkens the atmosphere in Summer, is nothing else than myriads of insects, so minute, as entirely to elude human vision. And the same naturalist has demonstrated, that these do not exist separately only, but also congregated in societies similar to those of bees and ants, and that these also observe amongst each a certain regular order.

From any evidence of our senses, therefore, we must draw no argument, as to the impossibility of the thing; for the divine providence of the all-wise creator of the universe is equally manifested in the most minute, as well as in the greatest, of his arrangements. Whilst we inspect a living acarus, we feel assured

* "Vide Hoffmann. Systema. iv. 122-3.

that, minute as it is, it is provided with muscles, nerves, veins, arteries, and viscera: and that all these, however small, are yet adapted to contain the most subtile fluids, calculated to circulate through them; all this too we are perfectly warranted in believing. Whence these animalcules shall have proceeded we may well wonder, but we are scarcely warranted in that wondering, if at the same time we but think that these being of such very minute proportions, that they hardly exceed in size the atoms of the atmosphere, can yet fly through the air and seek out the most minute chinks, exactly in the same way as mucor arises constantly from its seeds, sowing themselves wherever any putrid matter shall exist in a state fitted for their reception." 7.

Having very ingeniously applied this animalcular doctrine to various diseases, as scabies, dysentery, hooping-cough, small-pox, measles, plague, and syphilis, of which we shall take due notice in the promised analysis, we must here take a long leap, and come to cholera. Previously, however, to this, we shall probably be pardoned for introducing some consolatory considerations that may, in some degree, reconcile us to the sweeping pestilence that is approaching our shores. It appears that early in the 14th century a plague ravaged Europe, taking its origin in Asia, and destroying *only two-thirds of the human race!* It commenced in Cathay (upper Asia) in 1346, "from a most filthy smelling vapour, supposed to proceed from a certain fiery body which either fell down from the atmosphere, or was eructated from the earth." This vapour consumed all that stood in its way — animals, houses, trees, &c. for the space of fifteen days' journey in every direction.

"*And some most filthy little beasts furnished with feet and tails,* as also worms, and a small sort of snakes in a numberless multitude, fell at the same time from the atmosphere upon the earth, the stench and putrefaction from which, infected the very air, and all the regions circumjacent. A pestilence having arisen from thence, spread around, depopulating the whole of Asia, and afterwards Egypt, Greece, and Italy. Thence it passed into France, Spain, and England; and at length into Germany. In the city of Florence alone, Villanius says there perished 60,000, but St. Anthony says 100,000. Many prodigies are said to have preceded this plague in Asia, such as horrible openings and gulfs in the earth, exhaling a poisonous vapour, &c. (*Kircheri Scrutinium,* &c. p. 247.)" 171.

Dr. Neale supposes that these "filthy little beasts" could have been no other than "the rat-tailed larvæ of a species of fly, which inhabits cess-pools, and is known to entomologists by the name of *Eristalis tenax.*" That these larvæ do sometimes appear in most extraordinary multitudes, the Doctor can vouch, from what took place at Edinburgh in 1790, when he was a pupil at the high school there. We shall give this record in the author's own words.

"On a sudden, one day, there broke forth a swarm of these larvæ from some humid vaults that were connected with the necessaries, and the dissecting-rooms of the late Dr. Monro, the second of that name. These countless myriads proceeded, marching like an army down one of the wynds, or long alleys leading into the Cowgate, and having nearly reached the south bridge, turned on a sudden to the left, and proceeded along a rising street to the north, leading towards Hunter's Square, and the Tron church, where their career was terminated by the multitude (with staves and stones,) who came out to gaze upon such an extraordinary spectacle. The march of these larvæ continued, to the best of my recollection, for at least three days, and their numbers must have exceeded many millions; their line of march extending nearly to a quarter of a mile, from the back of the new college to Hunter's Square. Many persons must be now living who can remember to have witnessed this phenomenon." 172.

We do not wonder that our northern neighbours should have been energetic in the construction of a "New Town," where water-closets, and public sewers might supersede the domestic bucket and cess-pool!

But to return from this digression. The plague of insects occurred in China in the year 1347, while Edward the Third ruled in England, and various epidemic diseases soon prevailed over Europe, ending in a real plague. In London, such was the mortality that 50,000 bodies were interred in one week. The deaths at Norwich were nearly as numerous. Venice lost 100,000 people—Lubeck 90,000—while the deaths in the kingdom of Spain are said to have amounted to the incredible number of twenty millions!! In the following year the pestilence was still raging in the South of Europe, as well as in the North. In Denmark it assumed a novel form, and was called the "SORTE DIOD," or black death. The author strongly suspects that this is the disease now or lately raging on the shores of the Baltic, under the name of cholera. In 1358, Florence lost 100,000 citizens, as stated by Boccace in his wonderful description of that calamity. Petrarch asserts that very few escaped the malady.

Dr. Neale next comes to the subject of cholera, now "frightening our isle from its propriety," and threatening devastation to the people. A consideration of the phenomena, Dr. A. thinks, will induce us to conclude that "the efficient cause or contagion of cholera acts, in the first instance, on the brain and nervous system—just as the venom of the cobra de capello and the rattle snake, or any other deadly animal poison:—*that it is contained in the atmosphere*—enters the human body, most probably through the nostrils, which are always open, and is applied immediately to the brain, by means of the extremities of the olfactory nerves, gliding along these upwards through the cribriform plates of the ethmoid bone." The same might probably be said of the contagion of typhus and many other diseases. The following passage from the Englishman's Magazine is worth quoting.

"The entrance of the epidemic into Orenburg, took place on the 26th August, 1829, and its approach was attended with some extraordinary circumstances, which tend strongly to support the train of reasoning pursued in these researches. —'During the summer of 1830, the Tartars who frequent Moscow for purposes of traffic, predicted the approach of a pestiferous malady, which, however, the inhabitants, relying upon the local advantages of their city, would not credit. *Suddenly, however, the atmosphere was filled with dense masses of small green flies, which in Asia are the forerunners of pestilence, and are called plague-flies.* The streets swarmed with these insects; and as soon as the inhabitants quitted their houses they were covered from head to foot. *For a time, however, no attention was paid to this phenomenon, nor were any preventive measures against the cholera even thought of until intelligence arrived that this formidable disease had appeared in Nischid-Nowgorod.*'" 194.

The author can find no similar instance of such swarms of green flies filling the air, excepting in Plouquet's Bibliotheca Medica, under the article Pestis, where they are said to have appeared in Germany previous to that plague which raged at Nimeguen, during the time of Dimerbroeck. The author enters at great length into the investigation of animalcular contagion; but we are unable to follow him. Epidemic diseases are more speedily diffused and propagated during certain winds. In Europe, Asia, and Africa, it is during the sultry sirocco from the South-East. "In England, such a wind, in Spring, is generally attended by swarms of *aphides,* and is called *a blight or blighting wind,* by our farmers."

"Every one who has travelled through the south of Poland and Gallitzia, must have remarked, as I did in 1805, that the peasants there are carried on a bier to their graves, which are very shallow, wherein they are commonly interred without coffins. In cases of pestilence therefore, pestiferous flies and insects can easily find access to the bodies of the dead ; as they also can in Asiatic and Mahometan countries, and whoever has been obliged to pass a few nights in the houses of the Polish and Russian Jews, (the inn-keepers generally in these parts of Europe,) must have *felt cruelly,* how much they abound in all kinds of flies, bugs and vermin. In such countries, therefore, an epidemic disease is speedily disseminated, and soon believed to be infectious ; whereas in Hindostan and Eastern Asia, the natives being more cleanly in their persons, and bound by their religious ordinances, (Hindoos as well as Mahometans), to practise frequent ablutions ; clothing themselves also in white calicoes and muslins, (not in greasy woollens and decaying animal furs), pestiferous insects are more speedily destroyed, and cannot so easily nidify in their dwellings or about their persons. Hence, epidemic diseases, which in India are scarcely contagious, rapidly assume that character in Europe. Wine too being prohibited, and spirits little used, the Asiatics are more temperate in their habits of life, and gluttony and drunkenness, common vices in Europe, predispose the bodies of the poor labouring classes to receive such a disease as spasmodic cholera, from the over-saturating their blood with carbon and hydrogen, which are ever predisposing circumstances during the times of pestilence ; wherefore it has been remarked by the physicians of Russia and Poland, that the present malady chiefly attacks men rather than women or children, the poor and filthy, in preference to the affluent and cleanly ; and sots and drunkards especially beyond all others. What an additional motive and encouragement is this, for being cleanly and temperate ?— exemption from the attacks of pestilence and the increased probabilities therefore of a protracted and healthy enjoyment of life." 200.

The following passage will afford an entertaining proof of the extravagance to which a theory may be carried by an ardent mind. It is an account of an attack of cholera sustained by the author himself in the month of July, 1823.

"I was then travelling through Italy, on my route to Malta, and after two days of painful journeying from Rome through the Pontine marshes, had slept at *Terracina,* and next day reached *Mola di Gaeta ;* and while visiting the ruins of Cicero's villa there, was exposed to the disastrous influence of *a strong South East or sirocco wind.* The day was very sultry, and the journey in the afternoon towards the little village of *St. Agatha,* most oppressive. At *St. Agatha* I was roused from my bed about two o'clock in the morning with all the symptoms of cholera. A most severe storm of thunder and lightning was then passing through the valley. Fortunately for me, I had brought from Paris, an ounce bottle filled with strong laudanum, of which I took at intervals doses of about thirty or forty drops, joined with some Eau de Cologne, of which I had also a bottle in my travelling trunk. After four hours most severe suffering from cramp and oppression at the *præcordia,* the attacks of cholera ceased, and I fell into a state of syncope and collapse, in which I remained (as I afterwards learnt), about an hour, sleep followed and I awoke in a copious perspiration, and finding, on enquiry, that no medical assistance could be had nearer than Capua or Naples, I was assisted out of bed, carried into the carriage by the vetturino and a person of the inn, and, after great suffering, passed through Capua and finally reached Naples, where I slowly recovered. I consider that this was a slight sporadic attack of the true epidemic cholera for these reasons.—*First, that the*

*disease was then raging at Latakia and Antiocha, on the coast of Syria, on the eastern shores of the Mediterranean, and, upon inspecting the* Maps of the course of Cholera, *published by Dr. B. Hawkins and Mr. Kennedy, (the latter in the Englishman's Magazine), the reader will perceive on drawing a straight line from these regions to St. Agatha, that they lay directly east and by south of St. Agatha and Terracina.* Secondly, the matter vomited was the same glairy fluid resembling rice water, and the spasms about the abdominal muscles, diaphragm and region of the heart, resembled exactly those of the epidemic. I think I owed my recovery, under DIVINE PROVIDENCE, to the circumstance of my having fortunately the laudanum and Eau de Cologne at hand, and to my skin being in a perspirable state, from having been using warm baths at Rome for rheumatism in my knees and ankles. My convalescence was tedious, as I was afterwards attacked at Malta with dysentery, owing to the state of debility in which I had been left by the attack of cholera, and then drinking the tank-water of Valetta, *which at all times abounds with the ova of aquatic insects and animalcules, as has been already stated.*" 210.

The reader will surely smile at this *far-fetched* (in medical language we might well call it the *remote*) cause of cholera—namely, the animalcula floating in the air on the Coast of Syria, and wafted by the sirocco to Terracina in Italy, where they produced a "sporadic attack" of "*the true epidemic cholera!!*" How far "DIVINE PROVIDENCE" had any thing to do with the stock of laudanum and eau de cologne, laid in for the rheumatism, we will not venture to say. We are inclined to think that Dr. Neale did not witness the liquefaction of the blood of St. Januarius, and various other sacred and unquestionable miracles, in the Eternal City, in vain! Whether the heat and malaria of July, in the Pontine Marshes, together with the fatigue and excitement of wandering round the tomb of Cicero, near Mola de Gaeta, had any hand in the production of the Cholera at St. Agatha, it is not for us to determine. Being endowed with only plain John Bull reason and sense, we should not have travelled so far as the Coast of Syria for animalcula to excite an attack of cholera, under the above circumstances —especially if Dr. Neale quaffed a bottle of *Falernian* juice (and he surely would not have been so unclassical as to omit this libation to Horace) on Mount Massicus.

But we must take leave of this amusing and somewhat visionary volume, to which we have already dedicated more space, in this article, than was convenient. On the diffusion of epidemic diseases there is a chapter, in which Dr. Neale informs us that a philosophical friend of his suggested "that the minute insects, commissioned by DIVINE PROVIDENCE for this end, are probably wafted in straight lines through the atmosphere, in a similar manner to that pursued by storks, swallows, quails, and other birds of passage, but more especially by swarms of locusts, which last follow leaders who conduct their migrations." Dr. Neale thinks he has proved the truth of this theory by an occurrence which he witnessed in the month of August last. But we must refer our readers to the volume itself, page 234, for the particulars. We part from Dr. Neale in good humour, though not yet converts to his doctrine.

[MR. BELL.]

III. We next come to one of the best works that ever appeared on the subject of cholera, though not included in the list by the Leviathan of Literature, the Quarterly Reviewer—for reasons which he himself best knows, and which we can only suspect—we mean the work of Mr. Bell.* The able author of the volume before us served in India from the year 1818 to 1827, with unusual advantages for seeing the disease, under every possible aspect, in all its stages—in every variety of the patient's condition—in the hospitals, the camp, and in private dwellings—among the natives and the Europeans —among the newly-arrived and the seasoned. But even to all these advantages we do not attach so much importance as to the talent, philosophy, and impartiality, which every page of the work discloses. We therefore entreat the particular attention of our readers to the book which we are now reviewing—regretting that we cannot devote a whole article to it—but conjuring all medical practitioners to place it in their libraries.

Mr. Bell questions the propriety of the name given to this terrible malady. The vomiting and purging are merely symptoms, and far from being the most important features of the disease. The term *asphyxia* is much more appropriate. Thus the pulse may be gone, and peculiar secretions voided, before the victim is aware of having more than a slight indisposition. The following case will exemplify the insidious approach of cholera asphyxia.

"While the servant (a Hindoo) of Captain H. was bringing in breakfast, his master was struck with his appearance, and asked him what ailed him? he replied he had nothing to complain of but deafness, which he ascribed to sleeping in the cold night wind. His master, alarmed at his looks, sent him to the hospital tent (the epidemic was prevailing in the camp to which this gentleman belonged): on minute examination the man was found to have had some suspicious stools, his pulse had sunk, his skin was cold: He had Cholera, and became rapidly worse; and though he was put immediately under treatment, and was a man of good habits and strong constitution, he had a hard struggle for his life." 11.

Blood drawn during the progress of the disease is found, in the outset, to be dark-coloured—as the case advances it becomes thick, with a deficiency of serum, coagulating quickly, and showing no buffy coat. In the last stage of the disease the venous circulation is at an end, and the blood is so grumous that it can scarcely be forced out through a large orifice. The average time in which the disease ran through all its four stages was sixteen hours. Before entering on the ratio symptomatum of cholera, the author presents the picture of a case successfully treated, which he thinks "will greatly aid in the elucidation of the malady."

"A patient is brought into hospital in what has been called the third stage of the disease: his countenance is sunk; he has vomiting and purging; his skin is cold; his nails are blue; his pulse is scarcely perceptible; his breathing is oppressed, and he has spasms in the extremities. He is immediately placed in warm blankets; stimulants, including a dose of calomel, are administered; and a vein is opened in each arm, with the largest orifice. At first the blood flows very sluggishly, perhaps it is only procured by kneading the arms, but by and by the stream is more free, and as the blood flows it is improved in its colour; the patient feels the greatest relief, *the pulse rises,* and the colour of the blood testifies that the lungs are restored to their function. Little else is necessary; the patient has a second dose of calomel administered to him, is left

---

* Treatise on Cholera Asphyxia, or Epidemic Cholera, &c. By George Hamilton Bell, late Residency Surgeon, Tanjore. Octavo, June, 1831.

in a warm bed, and falls asleep. In the course of a few hours a cathartic is prescribed, to remove the colluvies which the restored secretions are pouring into the intestinal canal. It only remains to guard against local congestion and reaction; but in general, in a case treated as above, there is no such interference with recovery. The suspended functions seem to be at once restored: the blood is arterialized; the animal heat returns; the excrementitious secretions take place, and the kidneys recover their functions. And it is particularly worthy of notice, that even persons the most ignorant of the doctrine of diseases, who are at all accustomed to see cases of Cholera, are aware that the passing of urine by the patient, is an unerring test that the disease has been overcome." 23.

Such a case, he observes, must tend to shew that, in cholera asphyxia, the due proportion between the venous and arterial blood is destroyed. In all stages of it, the venous blood preponderates over the arterial. In the course of the author's extensive practice and patient observation, he became convinced that the hypothesis of *inflammation* would not explain the symptoms—neither would that of pure nervous debility—nor depraved secretion; for all *secretion*, in its proper sense, appears to be suspended.

"Here then was a disease in which, although there were great discharges of serous fluid from the alimentary canal, all the natural secretions were at an end, the animal heat had disappeared, the heart and arteries had ceased to act, and the blood in the veins was impeded or stopped; yet the sensorial and respiratory powers were little, if at all impaired." 32.

The following propositions embody the conclusions to which our author has come.

1. "The great ganglionic or sympathetic system of nerves, is possessed of a power wholly unconnected with cerebral influence, which it may retain after the brain and spinal marrow are removed, and which may cease to exist while these retain the full exercise of their functions.

2. To this system belongs the circulation and distribution of the blood; and it consequently has a most important share in regulating secretion, and in carrying on the involuntary functions. And,

3. To the suspension of this power of the system, is to be ascribed the disease which has obtained the name of Cholera Asphyxia." 36.

We wish we had space to follow our author through the various reasonings by which he arrives at these conclusions. They certainly are very ingenious, and highly probable. The reasonings and the cases tend strongly to shew that, "after cholera has put a stop to the circulation of the blood, the sensorial and respiratory powers retain their functions." Mr. Bell sufficiently proves that the fluids thrown out upon the internal surface of the intestinal canal are any thing but secretions. They are *exhalations* rather.

"The fluids evacuated by the stomach and bowels, during Cholera Asphyxia, are found to be portions of the component parts of the blood. In health we never see such fluids discharged from the abdominal viscera;—they are not gastric or duodenal juice,—they are not bile,—they are not excrementitious matter,—nor are they the mucous secretion of the canal. The Cholera discharge consists of the serum and fibrin of the blood. But then evacuations go on, and the bowels are filled, after the heart has ceased to act, when the arteries are empty, and when the capillary vessels are no longer supplied with blood by the usual course. The great veins, however, the liver, and the right side of the heart, are gorged with blood; the abdominal veins having no valves, regurgitation takes place, the capillaries are filled by a retrograde course of the blood, and are thus enabled to discharge the more attenuated parts of it. And although I believe that, in these circumstances, there is a deficiency of the nervous energy necessary towards *secretion*, still, as the medullary nerves retain their functions, the action by which this fluid is excreted, will retain the character of life, and will differ from the purely mechanical and chemical changes which take place after death." 58.

In respect to the effect produced by cholera on the cuticular discharges, Mr. Bell avers that perspiration almost invariably makes its appearance in the latter stages of the disease. Yet, at this time, it is almost impossible to produce vesication. The fact is, that inflammation cannot be induced on a surface where there is little or no arterial circulation. But we must pass on to—

### The Mode of Propagation.

The origin and progress of this disease, since 1817, is peculiarly interesting, " and gives a character to its remote cause differing from that of of every other disease." It travelled from north to south with surprising regularity, and apparently at about the rate of a degree of latitude per month. Thus it appeared at Ongole on the 14th of August, in north latitude 15° 30' and longitude 80° east—(Coromandel coast) and at Dharwar (Malabar), 15° 25' north, and 75° east longitude, on the 13th of the same month, August.

" It appeared in Soonda, in 14° 50′ N. and 74° E., in the beginning of September; at Hurryhur in 14° 30′ N. and 76° E., on the 12th September; and at Nellore, 14° 20′ N. and 80° 25′ E., on the 20th September. It appeared at Madras, 13° 5′ N. and 80° 25′ E., on the 8th October; and at Bangalore, which is 3000 feet above the level of Madras, in lat. 12° 57′ N. and 77° 46′ E., on the 22d of October. And as we approach Cape Comorin, on the extreme south point of India, we find it coming down one coast almost stage for stage with its progress on the other side.

We thus find the disease travelling from north to south, with unaccountable regularity; appearing in the line of 20° of northern latitude in the beginning of 1818, and reaching 8° north latitude on the 1st of January, 1819; holding its course, seemingly uninterrupted by winds, seasons, or climate; appearing at Dharwar on the 13th of August, in the height of the rains, when the thermometer seldom rises above 75° of Fahrenheit in the hottest part of the day; and at Ongole in the same latitude, but distant 5° of longitude, in the dry season, when the thermometer varies from 95° to 105°, within a few hours of the time at which it broke out at Dharwar. In the progress just referred to, far from being assisted by winds, the disease must have frequently travelled in direct opposition to their currents, and seems never to have been retarded or advanced by their direction." 73.

A map is given, shewing the steady and progressive advance of the disease from Ganjam, marked 3d March, 1818, to Palmacotta, 1st of Jan. 1819, during which time it traversed a space of between ten and eleven degrees of latitude with a surprising uniformity of pace—namely, as we said before, at the rate of 60 miles a month. We beg the reader's attention to the following passage, for reasons which will be sufficiently obvious.

" It may be said, that we are not to expect that a disease, which, like cholera, at once arrests the wayfaring traveller, is to pursue its course rapidly on land. But this objection to my argument will not hold on a sea-coast, where small trading vessels carry on uninterrupted traffic, for a great portion of the year, and where no quarantine laws exist. In such circumstances, a contagious disease would inevitably be speedily propagated along the sea-shores. And hence, instead of Cholera reaching the seaport of Madras, simultaneously with its appearance in parallel latitudes in the interior, some of the many trading vessels must have carried it speedily, from the tainted districts, to the seat of the Presidency, had the disease been capable of being conveyed by man or merchandize. The progress of Cholera down the Peninsula of India, when it visited that country in 1818, is so instructive, that I have thought it advisable to abridge the map published by Mr. Scott in his Report, already referred to. From this sketch it will be perceived, that Cholera, as an epidemic, was in 19° N. on the 10th March, 1818, that it travelled regularly at the rate of about one degree of latitude a month, and that it reached Madras on the 8th October in 13° N. This was its progress during the dry season, and when there was no interference with the constant commercial intercourse which prevails on the Coromandel Coast. On the 10th of October, annually, the port of Madras is closed, and in consequence of the prevailing winds, and of the surf, which, during the next two months, breaks upon the whole of that open coast, every vessel is forced to leave it, and the small trading vessels are drawn high and dry on land. By referring to the annexed map it will be seen, that far from this interruption to communication, retarding the progress of this extraordinary disease, it passed over the next five degrees of latitude, even more rapidly than over the former six; for we find it at Cape Comorin by the 1st of January, and it travelled in little more than a month to Negapatam, nearly three degrees south of Madras, during the height of the rainy season. This mode of progression can scarcely, I think, be explained on the principles of contagion." 81.

Its course through individual districts was, however, extremely eccentric. Often, instead of advancing directly to populous places, when prevailing on the principal road leading to them, it would make a complete circuit round villages, leaving them untouched till another time. After giving numerous instances of this capricious character of its progress, Mr. Bell observes:—

"These examples of the habits of the disease appear to me inconsistent with the notion of its being possessed of a contagious quality. For in a country where, as in India, no restrictive measures are adopted, a principal city in a district could hardly by possibility escape a contagious epidemic, when raging in its neighbourhood. An infectious disease, one attack of which affords no protection against another, which travels in the face of the wind, and prevails in all seasons, would spread in every direction from any point at which it had commenced its ravages; and we should not expect it to disappear until it had attacked every individual liable to suffer from it, and exposed to its pestilential influence.

The course pursued by the Cholera on its appearance in Russia, does not appear to have differed very much from its progress in India. It did not reach the Imperial territories when it was known to prevail on the great roads leading to Orenburg, where it at last broke out. And when it did commence its ravages at that place, there seems to be no reason to believe that it prevailed within reach. It does not appear to have spread directly; nor, although its general course was north-west, does it seem to have held that course undeviatingly; but at one time it capriciously passed over 50 or 60 miles, and then retrograded on the country which had thus appeared to have escaped; at other times, certain places appeared happily to be exempt, but, five or six weeks after it had left the country, it broke out anew in those very places which had had reason to congratulate themselves on their escape.

4. The annals of Cholera prove, that when it made its appearance in a camp or a city, far from extending to every habitation, it was almost invariably confined to particular portions of even the most populous places. Sometimes, in an army for instance, one or two regiments encamped together, or separated by other corps, were the only sufferers in an attack of the epidemic; one division, or even one street only of a town, had the disease existing in it; nay, its prevalence has been known to be limited to one side of a market-place. Removing a camp a few miles has frequently put an entire and immediate stop to the occurrence of new cases; and when the disease prevailed destructively in a village, the natives often got rid of it by deserting their houses for a time, though in doing so they necessarily exposed themselves to many discomforts, which, cæteris paribus, we should be inclined to consider exciting causes of an infectious or contagious epidemic. The bare statement of these facts affords a strong argument against the doctrine of contagion or infection, as a source of Cholera Asphyxia." 84.

Mr. Bell justly observes that it is no proof that a disease is contagious, because all the inhabitants of a place have suffered from it, or because it has attacked the attendants of the sick. This is a disease running so rapid a course that it cannot be carried to any great distance. It is supposed that medical men have suffered more than others of their rank in cholera; and this indeed we might reasonably expect, even if the disease were not contagious. Let us listen to facts.

"During the years from 1818 to 1822 inclusive, the numerical strength of the European troops in the Madras Presidency was kept up at 10,000 men. In the course of those five years 3664 cases of Cholera occurred in this force, of which 695 were fatal, or at the rate, per annum, of one casualty from Cholera to every 72 men. During the same period, the Madras medical establishment was kept up at 200 commissioned officers. In those five years, 33 medical officers were known to have had attacks of Cholera, of whom 13 died, that is, one in 77 per annum.—See Madras Report." 87.

The following passage will explain away one of the most triumphant arguments which the exclusive contagionists adduce in support of their doctrine.

"But it has been said to accompany troops, in marching into a district where it had not previously prevailed, and into which it has thus been introduced. This statement is so much at variance with the well ascertained habits of the disease, that it would require the most minute inquiry, and unquestionable evidence, to entitle it to credit. It has been repeatedly ascertained, that Cholera patients may be carried into hospitals crowded with patients labouring under other diseases, without these, or numerous hospital attendants, having the disease communicated to them. Yet it is asserted, that a regiment, travelling at the rate of six or ten miles a-day, has carried the disease along with it a hundred miles or more, communicating it to the inhabitants as it passed on! We have seen that a camp, by shifting ground a short distance, has put a stop to the ravages of the disease. And is it to be believed, that a regiment cannot get rid of it, with no cause but contagion for its continuance, by ten or twenty marches? I conceive, that this supposed proof of a contagious quality in Cholera may be otherwise accounted for. When travelling on circuit, I have found the disease prevailing in a district, before any report had been made of the fact, notwithstanding the most positive orders on the subject; and I am persuaded, that were any of the instances, adduced in support of the statement under consideration, strictly inquired into, it would be found, that the usual apathy of the natives of India had prevented their noticing the existence of the disease, until the fact was brought prominently forward by the presence of Europeans. It should also be borne in mind, that Cholera Asphyxia is not a new disease to these natives, but seems to be in many places almost endemical; whilst it is well known, that strangers, in such circumstances, become more obnoxious to the disease than the inhabitants of the country. Moreover, travellers have, superadded to the remote causes of the disease, fatigue and road discomforts, which are not trifling in a country where there are neither inns nor carriages.

The following extract from the Journal of my first journey in India, is illustrative of some of the peculiarities, in this disease, to which I have adverted. In July 1819 I marched from Madras in medical charge of a large party of young officers, who had just arrived in India, and who were on their way to join regiments in the interior of the country. There was also a detachment of Sepoys, and the usual numerous attendants and camp-followers of such a party in India. The Cholera prevailed at Madras when we left it. Until the fifth day's march, (50 miles from Madras), no cases of the disease occurred. On that day several of the party were attacked on the line of march; and, during the next three stages, we continued to have additional cases. Cholera prevailed in the country through which we were passing. In consultation with the commanding officer of the detachment, it was determined that we should endeavour to leave the disease behind us; and as we were informed that the country beyond the Ghauts was free of it, we marched without a halt, until we reached the high table land of Mysore. The consequence was, that we left the disease at Vellore, 87 miles from Madras, and we had none of it until we had marched 70 miles farther, (7 stages), when we again found it at one of our appointed places of encampment. But our camp was, in consequence, pushed on a few miles, and only one case, a fatal one, occurred in the detachment. The man was attacked on the line of march. We again left the disease, and were free from it during the next 115 miles of travelling. We then had it during three stages, and found many villages deserted. We once more left it, and reached our journey's end, 260 miles farther, without again meeting it. Thus, in a journey of 560 miles, this detachment was exposed to, and left the disease behind it, four different times; and on none of those occasions did a single case occur beyond the tainted spots." 91.

The instances where the mixture of cholera patients with others produced no extension of the disease, would fill a volume—and they were as numerous and remarkable in Europe as in India. Passing over Mr. Bell's observations on quarantine, we must make room for a short extract respecting restrictive cordons.

"A restrictive cordon assumes a different character. The effects to the sick, of a dread of contagion on the minds of their attendants, has been already hinted at. But its stanchest supporter admits, that contagion is but one of the causes of Cholera; while it must be evident to every one, that the remote cause of the disease pervades a portion of country, where it prevails for a time, and that all living, or coming within the bounds thus, for the time, marked out by the disease, are apt to be attacked. In a country free from sanitary restrictions, we have seen the inhabitants of infected spots find safety in flight. How different would their condition have been, had they been strictly hemmed in within the tainted limits. There cannot be conceived a situation more full of horror than a town, the site of which is infected with Cholera, and the inhabitants shut up in it.

The tendency, therefore, of admitting at once the existence of contagion in Cholera, in the present undecided state of the question, is injurious to trade, fatal to the sick, and fraught with melancholy forebodings to those who may be exposed to a visitation of the disease." 97.

We must now take leave of our author, and that with reluctance. Mr. Bell is an advocate for venesection in cholera.

"By bleeding in such circumstances we relieve the gorged vessels, and thus enable the weakened energies of the circulating power, to act on the disburdened organs of circulation, and to restore the current of the blood. The lungs recover their function, pure blood is thrown into the left heart, the arteries are again filled with fluid fit to support life : this, it may be supposed, reacts on the sympathetic system, and by and by its energies are completely restored. In this way only, can the effect of blood-letting in Cholera be explained. No other method will account for the almost instantaneous recovery which so often follows venesection in such a condition of the system as has been described—a recovery more immediate than that which follows the removal of mechanical pressure from the brain. And it is confidently asserted, that in no case in which it has been possible to persevere in blood-letting, until the blood flows freely from the veins, and its colour is recovered, and the oppressed chest is relieved, will the patient die from that attack of the disease." 105.

During venesection the patient should be laid in a warm bed, and the blood encouraged to flow by the horizontal posture. Artificial heat to the surface should be applied in every possible way. Leeches and cupping-glasses are also to be applied to the abdomen. Stimulants internally must go hand in hand with venesection. Opium, æther, camphor, ammonia, peppermint, brandy, and calomel are the chief medicines. Mr. Bell is more favourable to comparatively small doses of calomel and opium exhibited frequently, than to large ones at longer intervals. The patient has a most ardent desire for cold drink, and Mr. Bell, as well as some other experienced practitioners in India, found it at least not detrimental to gratify their feelings by cold lemonade. It is curious that although the surface is so cold and bloodless, the patient has a horror of hot applications externally or internally. A warm bath, even below blood-heat has been found almost insupportable. One thing is certain, that heat should be applied to the surface, with as little fatigue to the patient as possible. The difficulty of applying hot or vapour baths with celerity, is a great drawback to our therapeutics, and he who could invent a machine or means of applying heat to the surface of the body, with dispatch and economy, would deserve well of his country. Before we close the volume, we shall indulge our readers with one more short extract.

"When marching up the country with a detachment of troops in July 1819, a camp-follower was brought into my tent (9 o'clock, p. m.) in a blanket. He was reported to have been suffering from symptoms of Cholera during the whole afternoon. His skin was cool; pulse weak and thready; had spasms in the extremities; urgent thirst. I immediately bled him, and with some difficulty obtained a full stream. When about twenty ounces of blood had been removed, the man felt complete relief. Some brandy and water was administered, and he returned with his friends to his own quarters. He was able to accompany the detachment next morning." 119.

We cannot part from Mr. Bell without expressing the pleasure and profit with which we have perused his work, which ought to be in the hands of every practitioner without delay.

[Mr. Orton.]

IV. It is now time to turn to the work of Mr. Orton,* a gentleman who

* An Essay on the Epidemic Cholera of India. By Reginald Orton, Surgeon of the 34th Regiment of Foot. Second Edition, with a Supplement, November, 1831.

has had ample experience of the disease in India. This is the most systematic and extensive work which we possess on the subject of Indian cholera, and we regret that we cannot afford sufficient space for a regular analysis of the book; but still the importance of the subject, and the experience of the author, demand from us all the space we can possibly spare. While the disease was ravaging at a great distance, the minute symptomatology of cholera was not interesting to practitioners in this country, who contented themselves with reading the more prominent features, such as the spasms, the vomiting and purging, and the remarkable quality of the discharges. But now that the disease is amongst us, it becomes necessary to be thoroughly acquainted with all the symptoms of the malady, in its insidious approaches as well as in its most furious onsets. On this account alone, Mr. Orton's book is very valuable. The following graphic description should be well studied.

"The attack of this epidemic is usually sudden and violent, but, in a great majority of instances, not without some premonitory symptoms. It is frequently preceded by a simple diarrhœa, continuing several days, and still more commonly by other slight affections which are more characteristic of the disease. Most commonly it is in the middle of the night, or early in the morning that these ominous disturbances are first felt. An extraordinary depression of spirits and general uneasiness comes on, attended by tremor and sense of debility; giddiness and headach, and occasionally ringing in the ears, are also felt, particularly on rising from the recumbent posture, or making any sudden movement. Pains resembling those which attend the accession of fever are sometimes felt in the limbs. The bowels are griped occasionally, and natural loose stools occur; nausea comes on. The circulation and the temperature of the body are variously disturbed, but most commonly the pulse is accelerated and weakened, and the skin is moist, and colder than usual to the hand of another. These symptoms, or some of them, not unfrequently continue many hours, or even a day or two, without proceeding much farther, or exciting much attention.

In general, however, the severer affections quickly set in. The stools become extremely frequent and watery, of a peculiar unnatural smell, and greyish white colour, so as exactly to resemble conjee or barley water in appearance; vomiting comes on, and after the common contents of the stomach, a clear watery fluid, interspersed with flakes of mucus, is discharged; copious sweat breaks out; and the anxiety and the debility rapidly increase. The countenance assumes a very peculiar appearance, by which alone the disease may generally be distinguished. This is so remarkable as occasionally to render servants recognized with difficulty by their masters, even in the early stage of the disease.* It is difficult to be described, but it bears a striking resemblance to the appearance of *age:* and seems to arise from the paleness, wasting, and shrinking of the features, and the depressed and disturbed state of the mind, conveying into the countenance a strong expression of care, anxiety, and alarm.

It is usually during a fit of vomiting that spasms of the muscles are first felt. They are almost invariably of the tonic kind resembling common cramps; recurring in paroxysms of the duration of a minute or two, and at intervals of a few minutes, and attended with excruciating pain. They affect, occasionally and in turns, the whole of the muscles of voluntary motion, but particularly those of the legs and feet. In general they are far from extending at once over the whole muscular system, for they very commonly attack but one extremity at a time, or

* See "Reports on the Epidemic Cholera," published by the Medical Board of Bombay. Preface.

even only one muscle, particularly one of the gastrocnemii, drawing up its belly until it resembles a clenched hand in hardness, and almost in shape. The abdominal muscles and those of the chest are frequently affected, but not particularly so. In no instance have I had occasion to believe that the spasms were injurious by interrupting respiration or circulation. Great disturbances of these functions are indeed observed to accompany them, but these effects equally follow when the extremities only are affected. The returns of the paroxysms of spasm are frequently brought on by the slightest exertion; and in particular, they appear to be intimately connected, in regard to cause, with those of vomiting; for these affections frequently recur together. Great debility of the circulating system also very generally attends the accession of the spasms or severe vomiting. I have often found the pulse sink at once, on the first appearance of spasm in the muscles, from the natural state, so as scarcely to be felt, or even disappear entirely from the wrist in an instant; and in such subjects as are not affected with spasms (for they are not a constant symptom), this fatal failure of the circulation not unfrequently appears with the first paroxysm of vomiting. The frequency of the pulse is very variable. In many instances it is rapid from the commencement; in others its frequency is little changed in the early stage; but in all it becomes extremely quick as the disease advances.

The respiration from the first severe accession is observed to be hurried and oppressed, and is frequently complained of. As the disease increases in violence, the colour of the whole surface changes to a livid hue, particularly round the eyes and at the extremities. The surface is bathed in cold sweat; the hands and feet, and afterwards the whole body, rapidly grow cold. The plumpness and natural expression of the features disappear; they become sharpened—in short, the whole *facies Hippocratica* succeeds; and with a rapidity which is very surprising, for it is scarcely possible to conceive how so great an emaciation of the countenance can take place, as it frequently does, in an hour or two." 3.

And yet a small number only of the symptoms are comprised within the above description. An inextinguishable thirst is usually complained of; and although the body is cold, there is an ardent desire for cold water, which, when swallowed, affords no relief to the morbid sensation. An acute pain is felt in the region of the stomach, increased on pressure. The urine, in the early stage, is usually pale and watery; but, as the disease advances, the urinary and biliary secretions are suppressed. The hands are sodden with cold sweats, shrivelled and wrinkled, like those of a washerwoman. A strong and disagreeable odour, *sui generis*, is exhaled from the body. This odour can never be forgotten. The patient is not sensible of the great diminution of temperature in the body—on the contrary, he often complains of heat, and is constantly throwing off the bed-clothes. On the accession of the spasms, or thereabouts, the great struggle takes place, and is quickly decided. In a few hours a remarkable change appears, which might lead an inexperienced practitioner off his guard, and to form a favourable prognosis. The spasm, the vomiting, and the purging cease, usually about the same time. The stomach becomes retentive, and clysters are not rejected. A tendency to sleep occurs—but the state of the circulation, and the cold livid condition of the skin, together with the ghastly expression of the countenance, forbid all hope. Death, in fact, is at hand! When Nature alone, or assisted by art, is about to triumph, a sound sleep is the invariable precursor—and that, apparently, not owing entirely to the opium which has been taken. It happens at all hours of the day or night—even in the midst of noisy and crowded wards. The patient awakes relieved—has a bilious evacuation—passes urine—and afterwards has a considerable purging of black, green, or yellow feculent matters. The pulse rises—the skin grows soft and hot, with copious perspiration. The sensations are most comfortable. This re-action sometimes rises to a considerable height, assuming all the characters of the idiopathic bilious fevers of the tropics. In many instances, the fatal effects of the poison were instantaneous, and the patients dropped down as if struck by lightning, and quickly expired. The burning pain in the stomach, increased on pressure and the reception of food, is considered by Mr. Orton as indicative of inflammation, especially as the *post-mortem* appearances tend to confirm that inference, whenever the disease has continued long enough for that process to produce its proper phenomena in the body.

But there are great irregularities and anomalies in this terrible disease. The spasms have been often absent. Thus, in one report, by Mr. Conran, the epidemic was found to exhibit scarcely any instance of cramps or spasms, while all the other usual symptoms were present. The absence of purging was also often noticed. Dr. Girdlestone does not even mention this symptom. Vomiting, though one of the most constant phenomena, has been frequently absent. But we must refer to the work itself for an account of the various anomalies observed by the author and others.

We now come to the second chapter, containing the results of *post-mortem* examinations.

"A dark blue or livid colour of the surface of the bodies; existing in various degrees in different parts and different subjects, and most remarkable at the extremities. It appeared to prevail most in the more robust and sanguineous subjects, and in the more rapid cases. Blood taken from both the venous and arterial systems was found to be of a very unusual dark and purplish colour.

The internal organs in general were found much gorged with blood. This was particularly remarked in the veins of the mesentery and stomach, and in the lungs.

The stomach exhibited many extensive patches of a crimson colour, usually confined, in a great measure, to its inner coats. Similar appearances existed in the intestines, most remarkable on their inner surface, but appearing externally in a greater degree than in the stomach; they were much more evident in the small than in the large intestines, and often occupied large portions, or the whole of the canal. The degree in which these appearances in both stomach and intestines existed, seemed in general to bear some relation to the duration of the case, for in such as had been of several days' continuance they were highly marked.

The stomach contained the ingesta which had been given for some hours before death in considerable quantity, and little altered either in appearance or smell. Calomel was frequently found at the bottom of the fluid contents, and adhering in various places to the mucous coat. The intestines were in a great measure empty. Their contents were usually free from the slightest tinge of bile, and consisted chiefly of a dirty whitish mucus, resembling conjee or thick barley water, slightly tinged with milk. Great portions of them, particularly the large intestines, were frequently found contracted, so as with difficulty to admit the finger into their cavities.

The gall-bladder contained the natural quantity of bile, of no very remarkable appearance. The gall-ducts were found pervious, the bile flowing readily into the duodenum on pressure being made on the gall-bladder. No unusual accumulation of bile existed in the ducts or their ramifications; but a small quantity of that fluid, of the healthy appearance, might be squeezed out of each of the pori biliarii.

The urinary bladder was almost invariably found contracted to the size of a hen's egg, without containing a drop of urine; but smeared internally with a whitish mucus, similar to that found in the intestines. The ureters contained the same substance.

The veins of the brain were much distended with black blood. The minute arteries of the membranes of the brain were frequently found injected. These appearances of vascular action were most marked in subjects who had died perfectly comatose or apoplectic, and like those of the stomach and intestines in protracted cases." 42.

The inferences which our author draws from the post-mortem appearances are, that a high degree of venous congestion prevails generally in the internal organs—with a universal tendency to inflammation.

The third chapter is occupied with that thorny subject—the proximate cause of cholera. The circumstance, he observes, which strikes us most in this disease, is the prevalence of morbid phenomena throughout the whole system. No one organ is solely affected—hardly, indeed, can it be said that any one structure is more affected than another. The brain, the heart, the lungs, the digestive tube, liver, kidneys, skin, and even the muscular system, seem to be simultaneously stricken with the morbid poison. Arrest of the functions of secretion, and of the powers by which heat is generated, is one prominent feature of cholera.

"The conclusion to which these views, and others which shall subsequently be unfolded, appear to me irresistibly to lead, are the following:—

1. *That the proximate cause of cholera consists in a diminution of the energy of the nervous system.*

2. *That the deprivation of nervous influence thus produced, extends in various degrees to all the functions; and immediately produces the phenomena of the disease.*" 61.

On the above hypothesis, he thinks all the various phenomena of the disease may be accounted for. The author then goes on, in another chapter, to analyze and explain the various morbid affections of the muscular system, the secretions, the senses, the circulation, and lastly, the respiration and changes in the blood.

1. It is well known that profuse depletion by bleeding will induce convulsions. The cramps and spasms, therefore, of cholera, may well be attributed to privation of nervous influence from the brain.

2. It is now universally admitted that secretion is dependent on nervous influence. That great feature in cholera, the suppression of all secretions, is explicable only by the failure or diminution of nervous power. It is more difficult, in this mysterious disease, to account for the immense increase of fluids thrown out from the surface of the stomach and bowels. These fluids are, however, any thing but secretions. They are rather exudations.

3. The morbid secretions in cholera are numerous. Nausea, pain, heat, thirst, and anxiety, are referrible to the stomach; while vertigo, tinnitus aurium, headache, suppression of the senses, as of sight and hearing, appertain, of course, to the brain.

4. The sinking of the circulation is the most urgent, if not the most characteristic, of all the symptoms of cholera; and the rapidity with which it supervenes, in spare habits, is surprising. In a majority of cases, the pulse becomes small and weak on the accession of the more severe symptoms; but in the weakly natives, nothing is more common than for it to disappear entirely from the wrist on the appearance of the first fit of vomiting or the first loose stool. The author endeavours to account for this phenomenon and its opposite, inflammation; but we fear we have few clues to the explication of this and many other of the morbid phenomena in cholera.

5. The respiration and the changes of the blood next engage Mr. Orton's attention. The nervous system, though the primary mover, is itself subject to be controlled by its own subordinate agents. The brain cannot act without a supply of decarbonized blood from the heart, and it is evident that, in cholera, the decarbonizing process in the lungs is suspended, or greatly decreased. Our author thinks it extremely probable, that this failure of function in the respiratory apparatus is the cause of the diminished energy of the nervous system, which produces the other phenomena of the disease.

In the 5th chapter of the work, our author institutes an interesting inquiry into the relations of cholera to other diseases. The famous and fatal sweating-sickness is first examined, and Mr. Orton thinks it extremely probable that it was identical with cholera morbus. We are by no means convinced on this point. The following passage from Armstrong, however, is not a little curious, as extremely applicable to cholera.

> "Here lay their hopes (tho' little hope remain'd)
> With full effusion of perpetual sweats
> To drive the venom out. And here the Fates
> Were kind, that long they linger'd not in pain.
> For who surviv'd the sun's diurnal race
> Rose from the dreary gates of hell redeem'd:
> Some the sixth hour oppress'd, and some the third.
> Of many thousands few untainted 'scaped:
> Of those infected fewer 'scap'd alive;
> Of those who liv'd, some felt a second blow;
> And whom the second spar'd, a third destroy'd." 121.

Time has almost buried in oblivion the horrors of this mammoth of diseases; and it is to be fervently wished that its memory may not be harrowed up by cholera!

Mr. Orton thinks it will probably be found that some other epidemics, which have received the name of plague and pestilential fever, have been no other than cholera, in some of its various forms. Burserius classes the sweating-sickness under the head of *ephemera maligna,* and gives an epidemic of this kind which attacked the soldiery, but leaves us in the dark as to date.

> "Many were attacked at the same time; their faces became of a bluish yellow; their eyes were like those of a person half dead, sunk in the sockets; their nose and forehead became sharp, and their skin rigid; their superior and inferior extremities were at first pale, a little after they became cold, and successively livid and black; the pulse was very weak." 128.

An epidemic that ravaged Marseilles, in 1720, bears a still more striking similarity to the cholera; but we dare not dwell longer on this subject. The relation which cholera bears to the effects of poison is very remarkable. Orfila relates the case of a man poisoned by an ounce of tartar-emetic, and adds—"the irritation which it excited on the alimentary canal produced a set of symptoms which I compared to *cholera morbus.*"

The sixth chapter is on the remote causes of epidemics in general. We must pass rapidly over this chapter. Speaking of the various efforts which have been made to elucidate the remote causes of these fatal visitations, he observes—

"I cannot pretend to give an abstract of these researches, but the inference which appears to have been almost unanimously drawn from them, and which is at once the most obvious and apparently the only feasible way of accounting for these visitations, is, that they depend primarily on some disordered state or states of the atmosphere. Many other causes have indeed been adduced, that doubtless contribute to produce them, the principal of which is contagion: but it has been clearly shown, that that agent alone, in a vast number of instances, is totally insufficient to account for their prevalence: for, on one hand, it is found that they frequently appear when there is no sufficient evidence of the presence of contagion, and spread so suddenly and extensively as to preclude all explanations of that kind, though the importation of infected persons or the morbific fomites is proved; and, on the other hand, they are found not to spread generally and to cease though these causes are actually present." 159.

Mr. Webster, of America, has written two volumes to prove that the causes of epidemic diseases arise from the earth, and are diffused through the air. A short quotation is well deserving a place here.

"Mr. Webster believes that pestilence and earthquakes depend upon one common cause, which excites into action the internal fires: but he supposes the action or fermentation may precede for months, or even years, the explosion in earthquakes; and by means of insensible vapour, or heat, or electric discharges, the elements of water and air may be affected in such a manner, as to impair the principle of animal and vegetable life." 161.

Let us bear in mind what Sydenham says.

"It proceeds from a secret and inexplicable alteration in the bowels of the earth, whereby the air is contaminated with such effluvia as dispose bodies to this or that disease, as long as the same constitution prevails; which at length, in a certain space of time, withdraws and gives way to another." 163.

We must pass over three or four chapters dedicated to the etiology of cholera, with the exception of a short quotation shewing the author's opinions on contagion.

"Contagion, from many circumstances, is a cause whose agency it is more difficult to disprove; but, as far as general opinion goes, it is against the supposition of the disease being propagated in that way. All the evidence which I have obtained on both sides of this question, will be briefly stated *in a subsequent part of the work; and it will be found clearly against the opinion of its infectious nature.* The decision of the question is less necessary here, as it is admitted that contagion alone is inadequate to the production of an epidemical disease. But the influence of contagion in this epidemic may at once be entirely denied, on the sole evidence of its most extensive ravages on the first day of its appearance at a place, and its entire disappearance in the course of a few days; as in the instance already mentioned of the 34th regiment, and many others which might be adduced." 166.

Such is the opinion enumerated at page 165 of the work; but at page 313, we find a different view taken of the disease. He gives us his opinions in these propositions.

"*Prop.* I. The disease is contagious; that is, it is conveyed either mediately or immediately from person to person.

II. There is reason to believe that the virus which propagates the disease is of a very subtile or volatile nature, and is readily conveyed by the atmosphere; *whence it arises that there is little if any increase of danger from the most intimate communication with the sick during the prevalence of the disease, above that which attends the common intercourse of society.*

III. The latent period of the disease, or that which elapses between the application of contagion and the appearance of symptoms, is usually very short, and even sometimes imperceptible, but on some occasions it has been more protracted." 313.

It is abundantly evident that Mr. Orton has changed his opinions at a more recent period, and no man can be blamed for altering his opinions, from conviction. Mr. Orton does not, like too many others, give but one side of the case. He fairly states the facts pro and con, and is at full liberty to draw his own conclusions. From the passage which we have marked in italics, in the second proposition, our readers will perceive that the admission of Mr. Orton's doctrine can make but little difference as to sanitary cordons. The following passage is, at least, consolatory.

"The evidence of the anti-contagionists is necessarily of the negative kind. One of the strongest general facts which they produce is, that the numerous medical and inferior attendants on the sick in the hospitals have often entirely escaped, or suffered in a very trifling degree from the disease; and it must be admitted, that in a vast number of instances in India, those persons have suffered no more from their employment than if they had been attending so many wounded men. This is a fact, which, however embarrassing it may be to the medical enquirer, is highly consolatory in a practical point of view, both to him and to all whose close intercourse with the sick is imperatively required.

In Bengal, not more than three medical officers out of the whole list were known to have been attacked up to 1820, and but one died, though nearly the whole of them had largely witnessed the disease. This is a strong fact, which probably will have greater weight than Mr. Jameson's general assertions to the same effect:—that the medical observer in Bengal did not find his assistants or sick attendants more liable to be attacked with the disease than other persons; nor when one member of a family was ill, that the rest were more liable than others; and that when several of a family were attacked, it was in general simultaneously and not in succession. It is however more particularly stated, that in the Marquis of Hastings's camp, where the disease prevailed so severely, in no instance were the bearers of the sick—nor the immense number of hospital attendants—nor soldiers visiting their dying comrades—nor sick in hospital (with the exception of convalescents), attacked more frequently than other persons not thus exposed." 316.

Mr. Orton adduces the testimony of many medical practitioners, all of whom state that "their numerous attendants, though mixed with the cholera cases, either entirely escaped, or suffered but in a very moderate degree. And be it remembered that they were constantly employed in rubbing the patients for the removal of the spasms, placing them on the stools, in the baths, &c." Mr. Annesley, whose authority is so weighty, goes to the same point, setting aside the recent examples on the Continent. But these, and thousands of other facts appear to Mr. Orton, at page 320, "as no more than dust in the balance against those which may be opposed to them." This declaration, too, in the very same volume which denies contagion at page 166!! But the most astonishing circumstance is, that, at page 166, he refers to this "subsequent part" of his work, where we shall find the balance all in favour of the anti-contagionists!! We blame not, but admire, a man for changing his mind when urged thereto by conviction; but inconsistency and vacillation are very humiliating to the pride of science! The following is a fair sample of the proofs of contagion brought forward by Mr. Orton, and against which all other facts are considered as "dust in the balance"!

"And in the following year, the same officer reports, that a party had arrived there suffering from the cholera, which had attacked them on their march after exposure to *a heavy storm of wind and rain* on the Kistna, and destroyed about sixty of them before their arrival. The disease did not then exist in the station; but three or four days after the infected party arrived, it appeared in a detachment of artillery in barracks, two hundred yards in front of which the party was encamped. It spread to the native inhabitants and other troops, excepting the 30th regiment, which was in barracks at the distance of a mile; they entirely escaped. He also traces several cases in succession to close personal intercourse, and finds a medical officer and two dispensers of medicine attacked. He adds, that a medical officer, arriving a fortnight afterwards, reported that *the villages on the route from the Kistna were all suffering from the cholera, and that the inhabitants had stated to him that they had got it from that detachment.*"* 323.

We would be glad to hear how Mr. Orton accounts for the sudden breaking out of the cholera, after "the heavy storm of wind and rain on the Kistna?" Did it there arise from contagion? This is not attempted to be proved; but it is inferred, that a disease having once sprung up from natural causes, is always after propagated by contagion! But, after wading through various conflicting opinions, we find our author casting anchor in a temporary creed.

"We are therefore forced to the conclusion, however at variance with the common laws of contagion, that in this disease—at least in India—the most intimate intercourse with the sick is not in general productive of more infection than the average quantity throughout the community.

This conclusion is further strengthened by another hiatus in the evidence for contagion, *viz.* that though the importation of disease has so often been found immediately to precede its appearance in the inhabitants of a place, and even the first cases to arise in the neighbourhood of the imported virus, it has scarcely ever been possible to trace these cases to personal communication in any thing like a regular series, as may generally be done with the plague and other decidedly contagious disorders; and similar facts have been observed in much later stages of the progress of the epidemic. Since its entrance into Russia, it has more than maintained the contagious character which it exhibited in India. In numerous well-attested instances, the appearance of the disease in places previously healthy has been immediately preceded by the arrival of persons from infected places, and such persons have been the first to suffer. But here, as in India, it seems to have happened, that no sooner was the disease thus conveyed into a place, and perhaps a case or two seen in the new comers, or near them, than the thread of its course through the rest of the community was lost:—its progression from its origin till it gained head could not be traced. The deficiency of evidence on this particular point has been strongly adverted to in a valuable critique on the subject, which has appeared in the 108th number of the Edinburgh Medical Journal. It contains also a parti-

---

* Madras Reports, p. 127.

cular statement of the staff physician at Orenburg, showing, that, there at least, the disease had also exhibited that other marked anomaly which has been noticed, of the immunity of attendants on the sick. Dr. Smirnov states that his hospital attendants, twenty-seven in number, during the two months the disease prevailed there, when two hundred and ninety-nine patients were treated in hospital, entirely escaped attacks, although they were constantly employed in offices which brought them into the closest contact with the sick. So also the physicians in continual attendance, and various other officers of the hospitals, and even the washerwomen, all escaped.

The obvious inference from all these facts, and which can alone reconcile their contradictions, is, *that the morbific virus is of a most subtile and active nature, so that the atmosphere around the sick is quickly contaminated by it to a considerable distance, whence all who breathe such infected portions of the medium are equally liable to its influence ; and when it prevails generally throughout a city, the whole surrounding mass, though not equally, is sufficiently poisoned to produce the full effect of contagion.* From this *mobility* of the virus, and from the disease almost immediately following its application, it is enabled to spread with a rapidity, which, excepting in the instances of the sweating sickness and influenza, appears to be unparalleled in the history of epidemics ; and everywhere to strike its invisible but deathly blow where it is not warded off by insusceptibility : whilst in the great mass of people, who happily are thus guarded, probably the morbid secretions may be rubbed into the mouths of the absorbent vessels on the surface—or the effluvia arising from them inhaled into the lungs—or they may be taken into the stomach, or even inserted into the opened veins, without adding to the danger." 328.

By the way, the passage which we have quoted in Italics completely anticipates the celebrated propositions, or *official axioms*, promulgated by Drs. Russell and Barry. There is a remarkable discrepancy, however, between these claimants for priority of discovery. It is well known that Drs. R. and B. relinquish the idea of contagion by fomites. Not so Mr. O.

"Indeed it is not unlikely, that it is by means of fomites that the disease is in a great measure propagated over a country. During the existence of so acute a disorder, the patient is in no condition of conveying it from place to place ; and if his power of communicating it is confined to the actual period of its existence in his frame, which we must suppose, it must be very short." 347.

Here, then, the contagionists are at variance, one (the Board) maintaining that it is by the person, and not the clothes, that cholera is propagated—the other, Mr. O., conceiving that it is by the clothes, and not the person ! ! It is a fact, after all, that Mr. Orton adduces far more evidence for the contras than for the cons in this chapter, and that he seems to have altered his opinion, or doubted about the point where he was to fix his creed, throughout the whole of the investigation.

### ATMOSPHERIC INFLUENCE.

From attributing every thing to atmospheric influence, at one time, people are now running into the opposite extreme, and denying any operation to atmospheric changes. This has chiefly arisen from the circumstance of cholera having pervaded many climates of great diversity in their temperature. But these people should recollect that cholera has, in all ages, been the inhabitant of all climates, at some period or other of the year—and generally at a particular period. The present epidemic, though it has sometimes prevailed at unwonted seasons, yet has usually increased with increased temperature, and *vice versa*, both in India and in Europe.

The following propositions, with which Mr. Orton prefaces this chapter, are fully borne out by the various facts which he has collected from authentic sources.

"Proposition IV. Great irregularity and intemperature of the seasons has preceded and attended the rise and first spread of the epidemic over India.

V. The disease has prevailed in India chiefly during that half of the year when the sun was on the same side of the equator.

VI. A high atmospheric temperature is a great cause of the disease.

VII. A condition of the atmosphere indicated by the formation of thick clouds, heavy rain, and storms (particularly thunder-storms), or the immediate approach of these phenomena, is a great cause of the disease." 348.

It has been triumphantly adduced, as proof that atmospheric influence does not enter into the etiology of cholera, that "of the five camps visited by it, the centre division was attacked in the cold season, the Nagpoor and Sangur divisions in the height of the hot winds, and the Raja Pootana and Kurnaul divisions, whilst it poured down rain." Let us look at one of these divisions, the centre, and see what were the circumstances attending its visitation by cholera.

"It was an almost isolated attack, for the disease did not exist, at least to any extent, in the surrounding countries, so that none could tell from whence it came or whither it went. Nor did it subsequently spread in the neighbourhood, though five thousand persons deserted and dispersed themselves over the country. The intercourse between Allahabad and the camp was very great, yet the disease reached not the former place till four months after (see Mr. Spilsbury's paper in Trans. of the Med. and Phys. Society of Calcutta, August, 1829). Even some corps of the division stationed at a little distance escaped, though an infected party arrived among them from the main body. It was therefore at this time incapable of spreading beyond the camp by contagion. It is probable that the attack was owing chiefly to local malaria, combining with a high state of predisposition from the fatigue the army had previously undergone. Mr. Jameson states—'In the three grounds of encampment in which the disease prevailed most, the soil was low and moist, and the water foul, stagnant, and of brackish quality, and everywhere not more than two or three feet from the surface of the earth, and the vicinity abounded in animal and vegetable putrified matter; whereas at Erich, where the army regained its health, the situation was high and salubrious, and the water clear and pure from a running stream." 350.

The foregoing statement shews that if atmospheric influence were absent, terrestrial causes (to which indeed we attach much more importance) were in abundance. The visitation in question is pregnant with information also, as to the non-propagation of the disease by personal communication.

So far back as 1781, a body of 5000 troops were suddenly assailed by cholera at Ganjam, on the 22d of March.

"'Men in perfect health dropt down by dozens; and even those less severely affected were generally dead, or past recovery, in less than an hour. Besides those who died, above five hundred were admitted into hospital that day. On the two following days the disease continued unabated, and more than one half of the army was then ill.' It was at first attributed to poison, but it was soon discovered that the disease existed in the villages on their route, prior to their reaching Ganjam. There is no precise information of the state of the weather at the time, but it is stated that 'they had been marching almost incessantly for six days through sand and salt water; a violent wind blew day and night along the whole shore, and at night it was accompanied with such a penetrating moisture as to wet through the thickest woollen cloths.' " 353.

It appears that the year 1816 was exceedingly remarkable in Bengal for deviations from the usual course of the seasons. One earthquake occurred in April, and another in July. A great drought took place in the middle of the rainy season, followed by a deluge of rain, occasioning such an inundation as was unparalleled in the memory of man. These phenomena, however, were only accompanied that year, by an exuberant crop of bilious remittent fevers. It was in the next year that the epidemic cholera broke out.

" 'In February, 1817, the singular deviations from the ordinary course of the seasons which marked that year began, for that month had more the appearance of an autumnal than a cold weather month. It rained heavily every third or fourth day.' March was remarkable for thunder-storms, very heavy rains, cloudy alternating with clear weather, and southerly winds. 'The same kind of weather prevailed during the whole season, over that side of India, and from Loodianah to the Presidency, there was scarcely a district or village in which the prospects of the harvest were not blasted by the heavy falls of rain, and long-continued humidity of the atmosphere.' The two next months were not very different from those of ordinary years, the weather being very hot, and broken by occasional storms and falls of rain. The rainy season set in twenty days earlier than usual, and proved to be one of uncommon violence. 'In June and July there was hardly a dry day, and before the end of the latter month the river was quite full, and the country nearly under water. In the districts of Jessore, Backergunge, Nuddea, and every portion of the Gangetic Delta, there had been a long protraction of very heavy rain; and nearly the whole country, especially in the lower division of the province, was one sheet of water before the middle of August.' " 356.

It was about the end of May, or beginning of June, 1817, that the epidemic commenced in various places—though the exclusive contagionists find it very convenient to locate it in the city of Jessore, at a later period. It was in the district of Nuddea, not of Jessore, that cholera commenced.

" It is however shown that this was far from being its sole local source. Such and so striking being the circumstances attending the rise of the malady, and its first and principal ravages all over Lower Bengal, we may fairly infer that it was owing to this exaltation of the common causes of endemic or sporadic disease that it took on the epidemic and contagious form, and thus became capable of diffusing itself far and wide over the earth." 357.

Mr. Orton is of opinion that, as far as facts can be ascertained, the propagation of cholera through India, and afterwards through Europe, required favouring circumstances, similar to the above-mentioned, else it would not have extended its ravages so far and wide. It is to be confessed, however, that we have no sufficient data on which to ground any inference respecting atmospheric influence in originating or propagating the disease. The following historical facts are important.

" The influence of season on the disease during its progress through Persia and Turkey, appears to have been at least as evident as in India; for it appears from the statements of Dr. Rehman that in the whole of its course, for three years, from the shores of the Persian gulf to the Mediterranean in one direction, and to the borders of Russia in Europe in the other, it prevailed *only in summer*. It first appeared in these countries in 1821, at Muscat, a place so notorious for heat that it is termed 'Hell' by the Persians, and during a season of extraordinary heat. In the course of that summer it extended from Busheer as far as Yezd in the centre of Persia, where it ceased in the winter, and re-appeared at the same place early in the following year. At the same time it extended in the other direction from Bussora as far as Bagdad, where also it is lost towards the end of the year, and in like manner re-appears at Mosul, higher up the Tigris, in the following July. In that Summer it travels from thence to Aleppo, and from Yezd to Ghilan and Mazenderan, on the southern shore of the Caspian, at all which distant places it again ceased on the approach of winter. In June, 1823, it re-appeared in the neighbourhood of Aleppo, and prevailed to some extent on the Syrian shores of the Mediterranean; but in this direction its progress finally ceased in that year. In April, 1823, it also re-appeared in Mazenderan and Ghilan, extended along the western shore of the Caspian, and reached Astrakan in September. It had then attained the 46th degree of latitude, and its virulence seemed completely exhausted. It shortly ceased, after destroying only one hundred and forty-four persons in that city, and extended no farther. And as it ceased to advance for six whole years, the few who were watching its progress doubted not that it had at length reached its *ultima thule*, being incapable of existing in the more temperate regions of the north. But we were then, and probably still are, very imperfectly acquainted with the capacities, the habitudes, and strange varying features of this *Avatar of Siva*—which, like that stern deity, with his crest of snakes and necklace of skulls, seems to have a great propensity to *stalking round the earth*." 362.

The following passage, commemorating a sudden and awful outbreak of cholera in a marching troop, succeeded by a terrific thunderstorm the same night—a thunderstorm which the inhabitants of these Isles cannot form an idea of, is worth recording.

"The night of the day in which the disease appeared in a party of troops under my charge was marked by a thunder-storm. The anxiety and fatigue attendant on the occurrence, with perhaps a shade of personal apprehension, which the stoutest heart might be allowed to feel on such an occasion, were to be borne amidst that war of the elements, from which we were separated only by a few folds of cotton cloth; and, as if to add a climax to the scene, a flock of jackals kept howling, or rather wailing in their ordinary manner, all night round the camp; emitting in infinite variety of chorus, sounds closely resembling those of human beings in distress. It required no great stretch of the imagination to fancy for a moment, that they were the voice of the hydra-headed demon of the pestilence, mocking the sufferings of his victims!" 377.

But the influence of LOCALITY in the production and extension of cholera, is a more interesting and, we imagine, a more useful subject of investigation than that of aerial influence, over which we have no control, even if we had a correct knowledge. The following are the preliminary propositions of our author.

"PROPOSITION IX. The disease shows an evident preference for the low and level parts of a country, and avoidance of more elevated tracts.

X. It is found to prevail most severely in all the moist, close, and filthy parts of any city or neighbourhood.

XI. It commonly follows the course of rivers, both navigable and unnavigable.

XII. It prevails most severely in all those countries and places, where, from the prevalence of the endemic fever, malaria is known to exist; and in such situations its epidemic duration has commonly been greatly protracted." 401.

No sooner had cholera begun to emerge from the low and level territory of Bengal, than it was found to spread along the lines of country similar to those in which it was first engendered. It was repelled, at first, by the elevated plateau or first range of the Himalaya Mountains, running parallel with the Ganges. This was the case in other parts of India, during the first onset of the epidemic at least. "And not only were the elevated tracts and table-lands partially or wholly exempted, but the insulated mountains and hill-forts, as Khotass, Adjeghur, and Kallingur, which enjoyed an entire immunity, whilst in the plains all around, even to their very feet, the pestilence was sweeping off its tithe of victims." Unhappily this immunity was not permanent. The stupendous Himalaya alone proved a barrier which the malady was never able to pass. In its subsequent eruptions, it rarely failed to reach the place it had previously spared. The Neelgherry Mountains appear to have hitherto escaped. The remarkable attachment of cholera for the courses of large rivers is well known, and has been seized upon by the ultra-contagionists, as proof positive that the disease was carried along by human intercourse. They never stop to enquire whether this intercourse always exists, and whether other causes than contagion may be taken into account. India, which has furnished us with so much information on all other points, has also made known some curious facts bearing on this part of the investigation, which we would strongly recommend to the consideration of the exclusives. In Bengal, great traffic is carried on by means of the Ganges and its several contributory streams, and many of the great roads run along the banks of the rivers. Here the contagionists had fair grounds for supporting their doctrine of cholera by intercourse. But the peninsula of India also presented great rivers, on the banks of which the disease loved to dwell; but, unfortunately for the contagionists, there was little or no navigation intercourse on these streams, nor great roads along their banks. Hear what a contagionist (a contingent contagionist, we allow) says upon this subject.

"To what are we to attribute this characteristic of the epidemic? Its explanation, though not single, is very obvious—*human intercourse and malaria.* The great rivers of Bengal are also the great channels of internal communication, which is carried on either on their surfaces, or on the roads along their banks. The most populous places *all lie on the rivers,* and the intercourse between them must be very great.—No wonder that this especial visitant of man is found accompanying him on his journeys: such has also been the case on the great Rivers of Russia, and the fact has been traced to the same cause. *But in the peninsula of India the case is very different, for there navigation is scarcely carried on even to the most trifling extent on any one of the rivers, and scarcely an instance can be mentioned of a great road running on the bank of a river, for they almost all cross them; but the existence of the other cause, malaria, in these situations has already been made evident.*" 412.

The foregoing extract speaks for itself. In Bengal, "all the most populous places lie on the rivers—and where could we expect to find the disease most prevalent but in those situations where it had most provender? The same, we believe, will apply to the great rivers by which the disease entered Europe. But in the peninsula of India, where the rivers had no traffic and the banks no roads, the cholera appeared wherever there were people to work upon. Some curious instances of the partiality which this fiend evinced for certain localities, are contained in the following passage. All such instances are cautiously slurred over by the exclusive contagionists.

"In a large camp in Candeish, one corps at the *left* of the line was found to suffer extremely from the disease, whilst that which was at the *opposite extremity* entirely escaped. The *former* was in a lower and more confined situation than the other, the *latter* being situated between two hills, where there was a strong current of air. On the corps which escaped marching, another corps arrived and took its place, and enjoyed the same immunity, the epidemic still existing in other parts of the camp.* And that the exemption was not owing to want of susceptibility was proved, at least in one instance; for the first of these corps suffered very severely on its march after leaving the station.† The 53d regiment were situated in an airy and rather high situation at Trichinopoly, when the disease appeared: they did not escape, but they were attacked the last. It first appeared at Masulipatam among convicts in a *bomb-proof* (a chamber like a cave in the ramparts), and was for some time confined to that chamber, before it appeared in the rest. *It was different from the other bomb-proofs, in being ill-ventilated, crowded, and extremely damp.* Throughout it furnished more cases than the others. Some further time elapsed before the disease spread to the free population. Other prisoners in a dry and commodious jail, at the same time, suffered much less.‡ Two parties of European recruits arriving at Madras, one is sent into bomb-proofs in the fort, and the other into barracks at the Mount, eight miles off. The *former* is attacked whilst the *latter* remains free, and on being also sent to the Mount has no more cases.§ A corps encamped on low ground in very rainy weather was severely visited; of thirteen sepoys taken ill, six died. After a few days they moved to a higher spot, and *only one more case occurs*, which appears on the march to the new ground. During an attack of the epidemic experienced in April, 1823, by the 69th regiment in quarters, at the suggestion of the surgeon, the wing of the corps in which the disease prevailed the most was encamped on a piece of high ground in the neighbourhood, and he reports that *not a case occurred in that camp.*|| Mr. Mitchell reports, on a re-appearance of the disease at Palamcotta, 'It commenced its ravages to the north-east of the fort, and spread pretty generally through the small, low, dirty, and close houses in every direction. The hospital escaped its influence, probably because it stood on high ground and was very open all round, for certainly none of the sick, though upwards of ninety, where attacked. Only one person about the hospital was attacked, and he was in the habit of absenting himself from it at night.'

Dr. Henderson relates, that he was encamped with a division of the army in Burmah, in 1825, on jungly ground, when the disease suddenly broke out in the camp, fifteen or twenty persons dying of it in one day. On the following day the camp was moved to a higher ground a mile and a half distant, *after which not a single case occurred.* The same officer observes a particular barrack room in Fort St. George being exceedingly obnoxious to cholera (on one occasion many cases appearing in it and none in the rest), which was attributed to its contiguity to the ditch of the fort, the men's privies, and an offensive drain.¶ Mr. Jameson gives various instances of high and dry cantonments and other spots suffering little or not at all, whilst the neighbouring cities were severely scourged, and many more of close, filthy, and crowded cities suffering severely, whilst others of the opposite description almost escaped. Thus it was with Agra and Muttra, two cities on the Jumna, only forty miles asunder. At the latter place, which was severely visited, the extensive cantonments in its neighbourhood remained unaffected upwards of three weeks, whilst the disease was prevailing in the city, and then it began in their most distant part, nearly two miles from the city, which was on low ground near the river, and progressively extended to the

---

* Report, p. 89. † Annesley on Cholera, p. 215. ‡ P. 89.

§ P. 89, 37, 38. || P. 1. ¶ Searle on Cholera, p. 60.

other end of the line, which gradually ascended from the river. ' The Rajapootana force previously to the appearance of the disease was encamped on a ridge of sandy soil, sloping for a mile or two to a much lower tract. The grass was thicker to the left of the camp, where the declivity was too gentle to allow the water to flow off speedily. On this side of the line the disease broke out earlier, and was throughout more violent than in its right division. No cause could be assigned for this irregularity of action, except that in one part the tents were pitched higher, and were consequently drier than the other."* 415.

Mr. Orton admits that these and innumerable other facts are somewhat adverse to the doctrine of contagion; but endeavours to get rid of them by supposing, that contagion might be *imported* into the localities above-mentioned. He is, however, candid enough to impress upon his readers, "that it is not to contagion alone that we are to look for the origin of the epidemic; for, without contingent favourable circumstances, it (contagion) is perfectly harmless."

We have dedicated so many of our pages to Mr. Orton's work, that we must now be brief in further notices. In a chapter on susceptibility or predisposition, the following conclusions are come to, in the shape of propositions.

"PROPOSITION XIV. In every body of people there is a large proportion incapable of receiving the disease, though exposed to all its usual causes.

XV. Having undergone an attack of the disease confers a great degree of immunity, at least for a time, from its future attacks.

XVI. The indigent part of society, the old, the weakly or unhealthy, and the intemperate, are particularly liable to the disease.

XVII. The usual exciting causes of the disease are exposure to great heat, cold, or moisture, errors of diet, over-exertion, the depressing passions, and in general whatever tends to debilitate or disorder the frame." 433.

These propositions, as usual, are elucidated by numerous facts and histories, drawn from the observations of the author and others. No possible doubt can be thrown on any of them—if we except the 15th, respecting the degree of immunity conferred on an individual by an attack of cholera. We have been led to form a lower estimate of this degree than our author; but we need not stop to discuss the point here. It appears that the Chinese take a very calm and philosophic view of cholera and other pestilential visitations. They observe that "the pestilence knows its victims," and that, in an over-populated country, it is not a curse but a blessing; for it deprives of life chiefly those to whom it is of least value—the wretched, the sickly, the aged, &c. and very commonly those who are of least use to the rest of society!

In the section which embraces a comparison of the epidemic cholera of India with that of Europe, there are some judicious remarks. The identity of the Asiatic and European cholera cannot now be denied. But it is clear that the proportion of cases in which fever follows the choleric symptoms, is much greater in Europe than in India—and this may well account for the greater cause which we have for suspecting a contagious character in the European disease. Fevers of all kinds are now generally acknowledged to be capable of taking on a contagious or infectious character, under circum-

* P. 116.

stances of accumulated population, filth, and deficient ventilation. The fever of cholera may therefore, like that of dysentery, pneumonia and other diseases, assume the fearful and dangerous attribute which the above circumstances confer. Dr. Kier states that, of twenty cases of cholera, seven terminated in the cold stage, and thirteen in the hot stage. It is uncertain whether the fever springing from cholera be a low or typhoid affection, or the consequence of some topical inflammation set up during the choleric symptoms. Mr. Searle, it will be seen farther on, affirms that more or less fever succeeds most cases of cholera—a bilious remittent in hot climates, a typhoid fever in cold. Mr. Orton finally remarks:—" although there is little hope of our island escaping a visit, sooner or later, of the disease; I agree with Dr. Johnson in anticipating no great extent of mischief from its prevalence." We must now dedicate a page or two to Mr. Orton's therapeutics.

TREATMENT.

The most important point of all is the early employment of remedies. Sydenham maintained that opium was our sheet-anchor in cholera, and our author confirms the opinion of the English sage. " It is probable that a single dose of it alone, given at the very commencement of the disease, would be found, in a great majority of instances, to put an effectual check to its progress." He warns us, however, against an exuberant exhibition of this valuable medicine. In its secondary effects—perhaps in its immediate effects, when given in very large doses, it is, Mr. O. thinks, productive of that oppression of the vital powers, which so strongly marks the intense degrees of the disease. Solid opium he prefers to the tincture, as being less liable to be rejected. Four grains appears to Mr. O. to be a proper quantity for the first dose. If favourable symptoms do not appear, a diminished dose may be repeated at intervals of three, four, or six hours. It is on the first dose, if early administered, of this invaluable medicine, that we are chiefly to depend. Injections of the tincture are also recommended.

" It has been the general practice to combine the opium with scruple doses of calomel (a mode of treatment for which we are indebted to Dr. Johnson), and the best effects have been stated by many practitioners to result from that measure." 305.

Others, however, have demurred to this practice, and the author himself does not give a decided opinion. " A practitioner," says he, " for whose opinion I have the highest deference, and whose practice in the disease appears to have been more successful than any other which has come to my notice, has never given the calomel until after the favourable crisis." Stimulants, as brandy, aromatic tinctures, camphor, and essential oils, are strongly indicated in this disease, when judiciously administered, in moderate quantities; but a too long perseverance in them must be highly dangerous, on account of the fever or inflammation which ensues.

" Bleeding has been extensively practised in this disease on all the three establishments, and has received the highest encomiums. There is, however, reason to believe that the opinions of medical men are still divided as to its general propriety. My own experience and enquiries have been such as at once to impress me with a high opinion of its good effects, and to show the necessity of caution and discrimination in its employment; for though its effects have been in general favourable, in some instances they have appeared the contrary." 306.

It requires some nerve, indeed, to attempt to abstract blood from a corpse-like body, where the pulse is scarcely to be felt, and the skin cold. But it is to be remembered that the sufferer lies in a state of asphyxia, or one very much resembling it, and that we abstract blood rather to set the congested mass in circulation, than to diminish the actual quantity existing in the vessels at the time—in the same way that we pour out a small quantity from a full bottle in order that we may be able to shake up the rest, and put it in motion. This was the idea that struck the Editor of this Journal twenty-seven years ago; and it is curious as well as gratifying that a hint then thrown out, should have led to the adoption of a practice throughout India and a great part of Europe. The Editor cannot resist the introduction of the following passage, since few of the writers on cholera have been inclined to acknowledge the sources from whence many important points of their practice have been derived.

"In justice to Dr. Johnson it is necessary to mention, that his invaluable work on Tropical Diseases (which is in the hands of almost every practitioner in India) appears to have led to the adoption of bleeding in this epidemic. And the merit which accrues to him from this suggestion is of no common kind; for, according to the unfortunate notions which have so long prevailed, it is probable that few persons would have ventured on a practice of this kind, in a disease whose principal characteristic is extreme debility, without some previous evidence of its propriety." 307.

This operation should invariably be performed in the horizontal posture, and care taken to prevent the patient rising afterwards. In fact it is advisable to preserve this posture throughout the disease.

The various means of exciting counter-irritation on the surface are amongst the most valuable and least objectionable remedies. Sinapisms are most generally used. Our author often rubbed the part intended to be blistered, with oil of turpentine and sand, previously to the application of the blister. The stomach, abdomen, and head are the usual parts to be counter-irritated. Speaking of the warm air-bath, Mr. Orton strangely supposes that it must be injurious by vitiating the air which the patient breathes. He does not seem to be aware that the patient's head is entirely free from the bath. We shall say more on this subject towards the close of the article. The thirst being excessive, and yet the ingurgitation of fluids being productive of vomiting, our author has usually allowed moderate quantities of infusion of ginger, which, with the addition of a little sugar and milk, forms an agreeable beverage, of a light stimulant kind. After the favourable crisis, purgatives are indispensible for preventing or removing the train of fatal sequelæ which so frequently attend the disease. They are found to produce copious discharges of vitiated bile and fæces. "Calomel, from its known powers in this way, is highly useful; but it appears advisable, in general, to avoid its full effect on the mouth." The great debility which necessarily follows, for a short time, seems to call for stimulants and nourishing diet; but these are dangerous remedies. Simple debility is seldom dangerous in this or in any other disease. The food therefore, should be light and unirritating. Change of air has a remarkably beneficial effect during convalescence from cholera. This is the whole of the therapeutic portion of Mr. Orton's work—only about seven pages in a volume of nearly 500 pages.

In conclusion, we may remark that the first edition of the work, founded on personal observation, propounds an unequivocal and exclusive anti-contagion doctrine—while the supplement to that edition, constituting the second edition, shews our author converted by the reports of others, to the doctrine of contingent contagion. This fact, or rather this change, though it derogates not from Mr. Orton's candour or perhaps his judgment, will nevertheless diminish the weight of his authority. Had he formed his final creed from his own observations and reflections, during the period of his widest range of experience, it would have carried with it infinitely more force in the minds of others. But putting this aside, the work which we have just closed is perhaps the very best which we have on the subject of cholera—at least as far as varied and laborious research is concerned. Mr. Bell's is more concentrated—more original—and we believe equally useful.

[Mr. Searle.*]

V. This gentleman's larger work we reviewed in Volume XIII., p. 375, *et seq*. The present pamphlet was published in an imperfect state, while the author was at Warsaw, superintending a cholera hospital there, and is now re-published with additions and improvements. Mr. Searle's testimony on all points, both of etiology and practice, is very important, since few men have seen more of the disease than himself, both in Asia and Europe. We must pass over his symptomatology and pathology, in order to give some notice of his present ideas respecting etiology and practice.

"The immediate cause of Cholera would appear to be of the same nature as that which ordinarily gives rise to fevers of the intermittent and typhoidal character: hence it attacks those more particularly who reside near to or frequent damp marshy situations, or respire an atmosphere otherwise contaminated by the exhalations arising from organized substances, vegetable or animal, in a state of decomposition: hence, too, it is, that filthy, low, unventilated situations are its most common habitats. To develop the disease, or to render the individual susceptible of its attack, it would appear, however, that a certain condition of system or predisposition must exist; else it would be of more general prevalence than we ordinarily find it; this I believe to consist principally in debility; hence the indifferently fed, the badly clothed, and comfortless poor, or those exposed to the inclemencies of the weather and vicissitudes of temperature, and particularly, when under exhaustion from the want of food and bodily fatigue, are the most frequent subjects of its attack. And on this account, troops while marching, are very liable to be affected. The causes enumerated I consider quite equal to the production of the disease; but, in the character of an influential agency, I am constrained to add, a peculiar condition or epidemic influence of atmosphere; seeing there are times in which it prevails to a considerable extent—over-stepping the boundary we have marked out and assigned to it by predisposition—and other times again, when the causes enumerated would appear to be in operation, without its production. This atmospheric agency or modifying cause I believe, however, to be, without attempting to define its nature, though

* Cholera, its Nature, Cause, and Treatment, &c. By Charles Searle, Esq. Octavo, pp. 51. Highley, November, 1831.

I think it probably dependent upon a negative condition or peculiar electrical state of the air (or earth influencing the air) in character one, simply of depressing influence; which being superadded to the amount of causes which under ordinary circumstances are productive only of that degree of depression of system and cold stage of fever which, like the shock of cold water, eventually excites the system to the re-action of fever—is, from being thus multiplied in extent, analogous to extreme cold, productive of that overpowering depression whereby the energies of the system are rendered unequal to the development of the stage of excitement or febrile condition." 8.

The indications of treatment which our author points out are these—" the removal and superseding the action of a sedative poisonous agency upon the system, and the effects resulting therefrom." It is easier, however, to lay down indications than to effect them by remedies. Those recommended by Mr. Searle are, external heat applied in the recumbent posture—an emetic, composed of a large table-spoonful of salt in a pint of hot water—bleeding from a small orifice, if there be pain or heaviness of the head, oppressed respiration, pain at the præcordia, severe spasms, and the pulse be moderately full. He recommends great caution in the employment of this measure, with careful attention to the effects resulting from it. The next step (using heat and friction) is the exhibition of 12 grains of calomel, with some cordial stimulant—and this dose to be repeated every hour or two, according to the urgency of the case. As the symptoms improve, the dose is to be diminished, but still to be continued, till bilious stools and the urinary secretion are restored, when a mild aperient may be combined with it. The brandy and water may be continued every quarter or half hour, till feverish excitement becomes developed, when the patient usually calls for cold drink, which may be accorded, in small quantities at a time. When the febrile re-action is fairly established, calomel and James's powder are to be given, with the neutral salts to secure evacuations. Local inflammations are now apt to arise, and are to be carefully watched. Mr. S. strongly recommends, at this period, glysters of warm gruel and common salt, repeatedly thrown up. Large sinapisms to the epigastrium and abdomen are beneficial—cramps are relieved by heat and friction. If the patient is not seen in an early stage, and symptoms of collapse are setting in, our author advises 20 grains of calomel to be given, with some cordial drink, at once, and local inflammations to be met by local bleedings and counter-irritation.

In India, Mr. S. observes, cholera was generally based upon, or succeeded by, fever of a bilious inflammatory type—in Europe, of a low remittent or typhoidal character. In Europe, the choleric symptoms were less marked than in India, and the succeeding fever evinced less of simple re-action.

" I have said remittent, though the first few days I have generally found it to be intermittent; coming on daily at about the same hour, preceded by coldness of the extremities, quivering of the lip, and depression of the circulation: but from the excitement of inflammation, which but too frequently becomes developed in the organs previously congested, the intermissions become imperfect, and in consequence, it assumes a remittent; and, from the conjoint debility, a typhoidal form." 32.

Almost all the cases in Poland, which were neglected or ill treated at the beginning lapsed into this form of fever. This is a strong proof that the choleric symptoms are only a stage or form of fever. The following passage is important.

"In reference to the foregoing, and in exhibition of the connexion that subsists between cholera, fever, and dysentery, I would add the notice of a milder species of the disease, which was, in the month of August, exceedingly prevalent at Warsaw, and where fever and dysentery are, I was informed, annually at the same season extremely common. The following is the best account I could collect from my patients of its insidious mode of attack. A sense of fulness at the præcordia, of languor and incapacity to exertion—mental or bodily, occasionally with giddiness or headache; the latter, however, was often attended with an obscure form of fever, and only felt at some particular hour of the day; a slimy coated, white, or furred tongue, and which appeared occasionally to be swollen, being indented along its edges by the teeth; or otherwise, an unusually clean, smooth, and red tongue; lips pallid, or of leaden hue; eyes often of a pearly appearance, and surrounded with a brown circle; the countenance sallow; appetite frequently but little impaired, though the digestion in general imperfect, evinced by flatulence and distention after a meal. Bowels at first constipated, succeeded, however, in general, by relaxation, and this when attended with inflammation, terminating not unfrequently in bloody muco-purulent evacuations, or, in other words, in dysentery.

The preceding symptoms, fluctuating with the weather and contingent circumstances, may continue two, three, or more weeks; the individual feeling that he is unwell, but not attaching any importance to his condition, till the depressing influence of the atmosphere, preceding or accompanying wet weather, or an attack of indigestion, succeeding to the use of some improper article of diet—as potatoes, cabbage, sallad, or the like, or drinking too freely of some cold fluid, or fatigue, or exposure to the sun, or cold—develops the attack of cholera, coming on by purging, or vomiting, succeeded by cramps in the legs, lividity of countenance, cold skin, and feeble pulse:—a condition from which, if the patient recovers, is almost invariably succeeded by fever, of an intermitting or remitting type, coming on daily or oftener, and generally unpreceded by any very marked cold stage, further than a sense of shuddering, tremor, or quivering of the lip, and depression of the circulation. An attack of this kind, it must be obvious, is nothing more than one of fever, based upon torpor of function, and congestion of the liver and chylo-poietic organs: and attributable to the continued respiration of an impure atmosphere of a milder degree than ordinarily gives rise to Cholera, such as results from the imperfect ventilation of the town, and foul state of the drains: or in persons otherwise situated, from some swamp or filth in the neighbourhood of their abodes." 36.

It is pretty evident, from the accounts from Sunderland, that diarrhœa and dysenteric affections prevailed to a great extent, and we have little doubt that the "malignant cholera" was merely the worst forms of the above diseases, aggravated by an epidemic influence. In the mild premonitory forms of cholera, Mr. S. recommends, after an emetic, a couple of grains of calomel every night, with an occasional mild aperient—keeping to bed, and using diluents. When the evacuations have become healthy, in attacks of cholera, Mr. S. recommends the sulphate of quinine, to prevent or mitigate the subsequent fever. Several interesting cases, and much useful detail of treatment, are contained in this pamphlet, which will no doubt have an extensive circulation.

[Dr. Lefevre.*]

VI. Dr. Lefevre was appointed, in conjunction with several of his col-

* Observations on the Nature and Treatment of the Cholera Morbus, now prevailing epidemically in St. Petersburg. By George W. Lefevre, M.D. Physician to the British Embassy at St. Petersburg. 8vo. pp. 96. Nov. 1831.

leagues, to superintend a large, though not very populous district, during the prevalence of the late epidemic in St. Petersburg; and he also did duty in rotation at one of the largest of the temporary hospitals. He had therefore ample opportunities of seeing the disease, of ascertaining the best methods of treatment, and of observing its mode of propagation.

In respect to the contagious character of the disease, Dr. L. prudently declines giving a positive opinion till he has further experience. "As regards the present epidemic," says he, "such as I have lately witnessed it, I am bound to say that I have no rational grounds for believing it to be contagious." In its progress, says our author, from Tifflis to Moscow, it was observed "to move in a zig-zag direction, rather than in a regular line of march. It would pass by a town which lay immediately in its path, to appear in another, which it must have reached by a circuitous route." This is what is called by the ultra-contagionists, *travelling* regularly along the great roads and rivers!

"When it invaded a town, it followed the same law; touching at a point to fly off at a tangent, and appear at a widely separated part from that where it first commenced, leaving the intermediate spaces uncontaminated.

Such was observed to be its character when it reached St. Petersburg, where it was first announced officially on the 14th of June, as having appeared in the suburbs.

A few days sufficed for its dissemination over the capital; and this so widely and so generally, as in most cases to preclude all idea of mere connexion with infected persons being the sole cause of its propagation.

I was myself called to see a case upon the third day of its appearance. The patient resided upon the English Quay, a distance of at least three English miles from the place where it first appeared. She was an old woman of sixty-seven years, who scarcely ever left her room; she attributed her attack to indigestion, and died in less than twenty-four hours with all the symptoms of inveterate Cholera." 3.

During the Autumn of 1830, St. Petersburg was "surrounded on all sides by the cholera," but still it escaped during the Winter and succeeding Spring. Seven months elapsed since the disease was in the immediate vicinity, and the imperial city began to think itself insusceptible of the poison. People resumed their usual avocations and ordinary diet—regretted the privations they had undergone—resumed the use of fruits and vegetables which had hitherto been thrown into the streets—and even laid aside the fumigations of tar and chlorides, juniper and tobacco. The family receipt-book was laid aside, and Buchan and Thomas were replaced by fairy tales and travels in the East. Castor oil and opium fell to the usual price, and chemists and druggists were the only people with long faces in the joyous St. Petersburg.

In the midst of this oblivion of danger, the hydra-headed monster was *en route*, and the journals announced his awful advent.

"The friends to the doctrine of contagion saw immediately the full proofs of their belief realized, because they found the disease imported by a bargeman, in an infected barge, and from an infected station. Nothing could be more evident, nothing more conclusive.

Those of a contrary opinion disputed the point, and with some degree of plausibility. *If the disease had been so imported, why did not the man fall ill before his arrival at St. Petersburg?*

*How was it that none of his companions, exposed to the same causes, should have been attacked also?*

When, upon inquiry, it was found that within the space of three days the disease broke out in a dozen parts of the town widely separated from each other, the supporters of contagion awaited further evidence, and the anticontagionists increased with the increase of the disease." 7.

How exactly does the invasion of St. Petersburg correspond with the invasion of Sunderland! A bargeman in health, and with a healthy barge-crew brings the cholera to the capital of Russia—a merchant vessel, with a crew in perfect health, and not one of which crew ever had cholera, sails up the river Wear, and—in the course of a few weeks, communicates cholera to the dirty lanes of Sunderland! Oh, this admirable *post hoc ergo propter hoc!*

From the dread of contagion, says our author, "many deserted their posts in the hour of danger, fled from the city in the day of her trouble, and shut their doors to all who had communication with her." Contrast these blessed effects of the doctrine of contagion with the following facts.

"I am personally acquainted with a nobleman, who, upon hearing of the Cholera having reached Petersburg, left his country seat at a long distance from the capital, hurried up to town, and was to be found from morning till night acting the good Samaritan, with as pure and disinterested intentions as his prototype of old.

It is true he was a non-contagionist, and feared not infection, and this confidence adds much weight to the advantages of the doctrine, inasmuch as it stimulates the opulent to exert themselves upon such occasions with proper philanthropic feelings." 8.

We must pass over the symptomatology of the disease. Little mention is made of the peculiar urinous odour which some have perceived issuing from cholera patients. Indeed we are informed by Dr. Hamett, that this symptom was comparatively rare. Dr. Lefevre avers that during the epidemic in St. Petersburg, a general indisposition prevailed throughout the population.

Of all predisposing causes "moral affections were found to be the most frequent, and their baneful effects were not merely confined to rendering their victims more susceptible of the malady, but they produced a decidedly fatal influence on the constitution itself." Many died of pure fright in St. Petersburg, and we have no doubt that hundreds will die of cholera-phobia in London, should the disease invade us with much virulence. The following facts are worth a bushel of arguments.

"As far as my practice is concerned, both in the quarter allotted me, and also in private houses in different parts of the town, I have no proof whatever that the disease is contagious.

The first patient I saw was upon the third day of the epidemic, and upon strict inquiry I could not trace the least connexion between the patient, or those who were about her person, with that part of the town where it first appeared, a distance of several versts.

As regards the attendants of the sick, in no one instance have I found them affected by the disease, though in many cases they paid the most assiduous attention, watched day and night by the beds of the affected, and administered to all their wants.

I knew four sisters watch anxiously over a fifth severely attacked with Cholera, and yet receive no injury from their care.

In one case I attended a carpenter in a large room where there were at least thirty other men, who all slept on the floor among the shavings; and though it was a severe and fatal case, no other instance occurred among his companions.

In private practice among those in easy circumstances, I have known the wife attend the husband, the husband the wife, parents their children, children their parents; and in fatal cases, where from long attendance and anxiety of mind we might conceive the influence of predisposition to operate, in no instance have I found the disease communicated to the attendants.

As for many reports which have been circulated, and which *prima facie* seem to militate against the statement, I have endeavoured to pay the most impartial attention to them; but I have never found upon thorough investigation that their correctness could be relied upon, and in many instances I have ascertained them to be designedly false; so that as far as proof can be drawn from my own limited experience, I have none to offer in favour of Contagion." 34.

In respect to diagnosis, our author justly remarks that it is utterly impossible to determine the nature of the complaint till the symptoms are at their height—" when once the cholera physiognomy is present, a child would be able to recognize it." The watery nature of the stools is one of the least equivocal symptoms of cholera at the beginning of the disease. The mortality pretty nearly averaged one-half of the cases. In the beginning of the epidemic the medical practitioners lost eight-tenths of their patients. In the decline of the epidemic, a similar proportion was saved. Various remedies were extolled as specifics; but soon found to be often ineffectual. Venesection was, at one time, ordered to be employed by *Government!* Then sweating was praised, and various ingenious contrivances were brought forth for the purpose of raising that process. " Of internal remedies, calomel and opium were most in repute." But this was far from successful. Rhubarb and magnesia superseded calomel and opium, and was, in its turn, renounced for sub-nitrate of bismuth. This was believed to be almost a specific; but the accounts given of its effects by various practitioners would excite laughter, were it not that the subject is too melancholy. The following is the conclusion to which Dr. Lefevre has come in respect to remedies. " The epidemic cholera, upon its first invasion, baffles all attempts to conquer it; but it gradually loses its intensity, and, towards its decline, becomes as tractable as other disorders of the alimentary canal." Hot baths at first were almost universal, but soon fell into disuse, and proved actually prejudicial, on account of the fatigue experienced by getting in and out of them. Frictions with hot cloths, or bags of hot sand, were found to be the best substitutes. The horizontal position is desirable throughout the whole period of the disease. The vapour-bath was, upon the whole, beneficial. " In the present epidemic bleeding has been decidedly beneficial, if employed judiciously."

" Bleeding from the arm in the first stage, when the pulse is full and the temperature not reduced, is often sufficient to cut short the disease. The quantity of blood to be drawn should be but small; eight ounces will be sufficient to allow the remainder to circulate more freely and relieve the heart, and this will not too much exhaust the patient.

The blood is generally thicker than usual, highly carbonized, and forms a loose coagulum. I do not know if the blood of Cholera patients has been analyzed during the present epidemic. The patient usually feels immediate relief, particularly where the head has been much affected. He should be bled in the horizontal posture, and remain quiet for some time afterwards. The operation of medicines is generally much facilitated by a small bleeding.

The absence of the pulse is no prohibition to the use of the lancet, unless this is accompanied by other symptoms of great debility, and the system has been exhausted by previous evacuations, and the surface is covered with a cold clammy sweat; in such instances I have never seen blood-letting serviceable, though many assert the contrary. In some cases the pulse ceases to beat very early, but upon opening a vein the blood flows slowly at first, gradually the current becomes fuller and stronger, the pulse beats very sensibly, and the heart thus relieved is enabled to continue the circulation." 57.

Dr. Lefevre makes some judicious remarks on the different remedies, internal and external, which require no particular notice. It appears that many of the poor people, believing themselves to be poisoned, took large quantities of milk and oil, which acted as emetics, and it is said that many of them recovered under this domestic treatment. When emetics were employed by the faculty, they were not so beneficial, in consequence of their being taken at a much later period of the complaint—and probably, also, from the patients not having any confidence in the remedy. The truth is, that at the onset of the disease, and, consequently, when it was most fatal, no remedy could boast of much success. When the violence of the epidemic was subsiding, then remedies acquired fame to which they were not entitled. Upon the whole the subnitrate of bismuth was considered a valuable medicine, though far from being a specific. The following was Dr. Lefevre's own mode of practice, when called in at an early period of the disease.

" If the patient is robust, the pulse still perceptible, and the system not too much reduced by evacuations, I order from six to eight ounces of blood to be drawn from the arm, the patient being first put to bed, in the recumbent posture.

The following draught is then to be given:

Laudanum and æther, of each twenty-five drops. Strong peppermint water, an ounce and half.

If this be rejected, it should be repeated immediately; if the second be likewise not retained, then a clyster of linseed tea with fifty drops of laudanum should be administered.

It often happens that the patient after taking the first dose falls asleep, and wakes in perfect health.

A large sinapism to the abdomen, and bottles of hot water to the feet, should not be omitted; if these means produce speedy relief, an ounce of castor oil should be prescribed as soon as the stomach and bowels are quiet." 80.

Such was the most successful practice in slight cases, and Dr. L. thinks that, by it, many of the severer forms have been warded off. But we have overstepped our limits, and must take leave of Dr. Lefevre. We are anxious to glean practical matter from the writings of those who have actually witnessed the disease, rather than polemical discussions from library practitioners and fireside travellers, who have never " set squadron in the field."

[Dr. H. Young.*]

VII. As it has been our object, in this extended article, to take notice

* Remarks on the Cholera Morbus, &c. By H. Young, M.D. Octavo, pp. 78. November, 1831.

only of a few of the more important and practical works on cholera, rather than crowd our pages with a galaxy of authorities, few of them of any real utility, so we must not pass over the unostentatious but valuable little pamphlet of Dr. H. Young.

The author had extensive experience of cholera in India, and transmitted the substance of this Essay to St. Petersburg in the early part of the present year. In his retirement after the fatigues and dangers of Indian service, the author would have remained silent on the subject of cholera;—

"Were it not that he dissents from the opinions promulgated by the Board of Health, as to the contagious nature of the disease—opinions which, if acted on, may, by giving rise to unfounded, or, at least, exaggerated alarm on this point, occasion the defection of friends and relatives, at a moment when their kind offices are of the utmost importance to the comfort of the patient, and when the absence of such affectionate attentions, by producing depression of mind, would materially diminish the chances of recovery."—*Preface*, vii.

It is now hardly necessary to state, that the Board that gave issue to the first code of health no longer exists, and that a new Board, convinced by their own reason, or warned by the public voice, has promulgated doctrines more in accordance with humanity and with modern knowledge. We shall be able to make room for only two or three extracts from this valuable little pamphlet, on account of our narrowing limits.

"It is a singular fact, that, throughout the whole of its progress, there was always a tendency shown by the disease to spread from east to west. It was natural, therefore, to look for some explanation of this phenomenon in the prevailing course of the winds at that period; and it so happened, that, in a great majority of instances, and in almost every situation, it was found that the wind was blowing either from the east, or east by south, at the time of the breaking out of the Cholera; and it was also observed, that the course of the winds had a very marked and decided effect on the violence and progress of the disease; for, while it raged with peculiar activity during the prevalence of an easterly wind, it was frequently observed considerably to decline, and, in some instances, almost to disappear, on the setting in of a northern or western one." 47.

"There was a marked disposition in the Cholera to follow the course of rivers, and this circumstance may, perhaps, be explained on two different grounds; first, it is to be recollected, that, in general, the rivers in India flow through a low and level country, subject to periodical inundations, and to copious depositions of mud and slime, on which a tropical sun acts powerfully the whole day. Hence, in all probability, they must prove the fertile source of miasm. In short, all the circumstances that predispose to the reception of the Epidemic are, probably, to be found in their neighbourhood. Secondly, the banks of the Ganges, and other sacred streams in India, are crowded with towns and villages, giving rise, throughout their whole course, to a dense population; and, as many sacred spots on their banks are, besides, resorted to by pilgrims from every part of India for devotional purposes, these circumstances may very readily account for the disease arising, and again and again re-appearing, in the course of large rivers. It is, at the same time, difficult to account for a circumstance which was in many cases very observable, *viz.* that the Epidemic would occasionally commit its most dreadful ravages on one side of a river, or even of a narrow stream, and leave untouched the opposite bank, although both were apparently placed under precisely the same circumstances; and then, after travelling a longer or a shorter course, it would return and exert all its baneful power on the places which it had before so unaccountably spared. Then, perhaps, after subsiding or disappearing altogether, it would, sometimes, without any assignable cause, return to a place it had before visited, and again commit the most deadly havoc.

That the severe visitation of the disease, in the course of rivers and among a dense population, may be explained by the causes above noticed, is rendered further probable by the same phenomena having occurred under the author's own observation, in the city of Calcutta and the adjacent villages. Many of the latter are placed in low, swampy situations, and, at that period, also, many of the most crowded parts of the city were very imperfectly drained. There were, likewise, in the lowest and most thickly inhabited quarters, many pools and ditches of stagnant and filthy water, on the margins of which it was the custom of the natives to sleep during the hot and sultry nights. In such situations the Cholera raged with double and dreadful fury, while the drier, better drained, and more thinly inhabited parts of the town, suffered, comparatively, very little." 50.

Upon the whole, Dr. Young concludes that the cholera owes not its existence to particular states of the atmosphere, but to a peculiar and active poison, engendered we know not how. But, however generated, Dr. Young thinks that the virulent matter is conveyed from place to place by certain currents of air, particularly by easterly winds. The following extract will shew our author's ideas respecting the contagion of cholera.

"In the first place, *according to the Bengal Official Report, this disease arose in many different places at one and the same time, and within a very few days it was raging in the unconnected and far-distant districts of Behar and Dacca.* Now it is by no means probable, but, on the contrary, against all experience, that it should have been communicated from place to place in the space of a few days only, through the many hundred miles that intervene between these districts; for the result of a number of observations shows, that in its progress from one place to another in any particular course, as, for instance, the course of a large river, its average rate of travelling, as marked by the successive periods at which places were affected, did not exceed five miles a day, and was very often still slower.

It differed from small-pox, plague, and other contagious diseases, in this, that, whenever it broke out in a town, camp, or cantonment, instead of daily increasing, and being perpetuated by the very means on which it fed, it invariably ran, within a given time, a regular course of increase, maturity, decay, and extinction. This peculiarity the author witnessed in the instance of the city of Calcutta, where the Epidemic broke out in the month of August, 1817, increased in strength and virulence during the months of September and October, began to subside from the beginning of November, and had almost disappeared by the end of January, 1818.

But, what happened in many other situations occurred here also; for, after an interval of about three weeks or a month, the disease re-appeared with more than its pristine violence, and again ran through the same course of increase, decrease, and decay. Now, as the author of the Bengal Report very justly observes, this uniformity of rise and declension appears to be quite inexplicable upon the supposition of contagion; for if the virus were capable of reproduction, through the medium of effluvia, or the secretions of individuals already infected, it must have gone on augmenting, until it either had no longer subjects upon whom to exercise itself, or was stayed by some powerful counteracting means, such as uncongenial seasons, or segregation, and the other prophylactic expedients usually resorted to on such occasions. This, at least, is the course commonly pursued by the plague and other contagious diseases. When once these are unfortunately introduced into a city or tract of country, they not only for a time remain prevalent in it, but go on daily increasing and perpetuating themselves by fresh accessions of infectious matter, until they either have depopulated the place, or are checked by some of the circumstances mentioned above. If the form and progress of the Epidemic Cholera had suggested the expediency of similar safeguards, they would, no doubt, have been proposed and acted on wherever it appeared. But, excepting the step wisely adopted in some of the camps in which the disease largely prevailed, of moving from the vicinity of the dead in quest of higher ground, and of a purer atmosphere, (a step which could have placed no check upon contagion, as most of the sick, and all the infected baggage accompanied the main body,) no means of security whatever of that sort, seem in any case to have been thought of: the truth is, that all men were convinced that they were wholly unnecessary.*" 55.

We are aware that it is next to useless to state that the Board of Health, where the epidemic first appeared, acknowledged that cholera broke out simultaneously in various and far apart places in Bengal. It commenced, say the Ultras, in the city of Jessore, and from that point it radiated, as it did from the barques on the Wolga, to every point of the compass, by personal contagion! The following passage will be read with interest by impartial men; while the ultra or exclusive contagionists will tear it up and throw it into the fire.

"On the morning of the 11th of May, 1818, a detachment of ninety men of the first battalion of the 26th native infantry, marched from an inferior post to join the main body of troops then encamped at Saugor. After an ordinary march it halted, in perfect health, half way, under shelter of a few trees, on the banks of a small lake, situated in the midst of an open space about three miles in circuit, and surrounded by low, woody hills. The whole remained well till the fall of night, when Cholera broke out amongst them. The first man was taken ill at midnight, and died in half an hour. Several others fell sick within the next few hours; and, before sun-rise, twenty out of the ninety were overtaken by the disease. Although the Saugor camp was distant only five or six miles, yet the detachment was too weak to move without assistance. The sick of the sepoys and followers were therefore carried in carts and doolies or litters, sent from the main body; but before eleven, a. m. when they got to their ground, five were already dead, and two others moribund. Next morning a man of the same party was seized in the act of scouring his accoutrements, immediately became insensible, and expired in a few minutes. During the three succeeding days several others were taken ill, and before the end of the week, of the whole detachment there was not a single man that was not sent to the hospital labouring under Cholera. The men of this party *mixed promiscuously* and *unreservedly* with those of the Saugor camp, and yet, of the latter, *not one individual got the disease*." 57.

Of a medical list, in Bengal, amounting to nearly 300 individuals, most of whom had to attend the disease on a very extensive scale, only three caught cholera, and only one died.† Anxious, from long experience, to mitigate the sufferings of those who are affected with this terrible scourge—remembering "that amongst the symptoms of the disease, was a remarkably acute sensibility of the mind, which, for the most part, continued to the very last moment of existence—and that the patient was, throughout, peculiarly susceptible of painful mental impressions"—the author feels that he should be wanting in his duty to the public, did he not "state his decided opinion of the non-contagious nature of the spasmodic cholera," and to endeavour to avert from the unfortunate sufferers the great calamity which would result

* "Bengal Report, page 127, et seq." † Bengal Report, p. 129.

from the adoption of the measures propounded by the first Board of Health in this country, viz:—

"'The immediate separation of the uninfected from the sick,' by their 'prompt removal from the house of any infected person, or by the removal of any individual affected with the disease; or, in the event of such removal not being practicable, the prevention of all intercourse with the sick, even of the family of the person attacked.'"*

Dr. Young's fears on this subject are now at an end. We shall neither be torn from our suffering friends—nor shall the afflicted members of our families be dragged to a lazaretto, to die of anguish rather than of the disease! Dr. Young's prophylactic measures are judicious, and his modes of treatment are active and efficacious. The grand measure is to restore the balance of the circulation, by external heat and friction, conjointly with diffusible stimuli internally. When the cold state is a little on the decline he recommends, from ample experience, large doses of calomel and opium. Bleeding did not succeed under his management.

We recommend Dr. Young's pamphlet to the attention of our brethren.

### VIII. MISCELLANEOUS WORKS ON CHOLERA.

It cannot be expected that we can give an account of the various works, of all sizes and shapes, that have been written on cholera, even since the date of our last number. We regret that Dr. KENNEDY's Notes on Cholera escaped us till too late to be embodied in this article. Dr. K. likens the proximate cause of cholera to CONCUSSION OF THE BRAIN—or rather he believes "that the collapse in cholera is induced by CONCUSSION of the BRAIN produced by some unknown cause, and that the purging and vomiting are sanitary processes." Dr. K. attributes the original cause of the disease to some peculiar state of earth or air, or both, and believes that planetary influence has a great share in the production of this cause. He strongly recommends venesection.

Dr. ALLEN, in his volume on Insanity has dedicated a considerable space to the subject of cholera. He is of opinion "that in all epidemics we have striking proofs of some noxious agency emanating from our globe, and which obeys some order of operation yet undiscovered—that it travels for the most part, with the sun, and its march appears subject to the same order of operation as that of magnetism:—that it will, like a thunderstorm or the whirlwind, march in some given line, often against prevailing winds; and, out of that line, we escape its baneful influence." This poison, which he believes to emanate from the earth, is received through the medium of the lungs, and from the lungs, passes to the brain, where it poisons the springs of life.

BARON LARREY has contributed his mite to the elucidation and treatment of cholera. His Essay is well translated by Mr. H. Patterson, of Dublin. The Baron has some curious notions respecting the nature and origin of this disease. He thinks it "consists in an aberration of the bile, and of the sero-albuminous portion of the blood. These fluids accumulated in the intestines, injure by their quantity, and above all by their stimulating quality. This irritation once established, upon the tunics of these organs, they almost immediately experience a spasmodic or nervous contraction, which is instantly followed by an antiperistaltic movement, &c." When the disease is epidemic, "it is the result of

---

* "Report of the Board of Health, p. 36."

the pernicious action of sudden transition from great heat to a low and moist temperature." "To this may be added at the same time, a greater or less mass of mephitic effluvia arising after stormy rains, from soil where putrid animal matters have lain, such as burying-grounds, dried marshes and stagnant water; and should hot winds afterwards arise and blow in the direction of habitations, the evil is aggravated and spreads rapidly." Such is the etiology of Baron Larrey. It is curious that he states the occasional existence of *exanthemata* in the third stage of the Indian cholera, and "this is the time when contagion may take place." We have heard, on good authority that eruptions on the skin, of a very remarkable kind, appeared in some cases of cholera along the shores of the Baltic. There is here given an extract of a letter from Dr. Vivier, one of the most talented physicians who went to Poland to investigate the cholera. "Whatever contagionist-physicians have said, nothing is less demonstrated, in cholera, than the property of propagating itself by mediate or immediate contact with the patients." We need not advert to the mode of treatment recommended by the venerable Baron.

Dr. Granville has sent forth a clever little volume, under the quaint title of "the Catechism of Health," a considerable portion of which is dedicated to the subject of cholera. In this portion he has applied "caustic to our councils," and handled the first Board of Health very sharply. Dr. G. is a decided anti-contagionist, and has brought forward some curious facts not generally known in this country. We shall allude to one or two of these. In 1821, an expedition sailed from Trieste, under Baron Schimellpenning, for the purpose of circumnavigating the globe. A spontaneous cholera broke out in the squadron, when it arrived in the hot latitudes, and destroyed nearly the whole of the crew. In the year 1600, cholera, in its worst form, made the tour of Europe, and destroyed a large proportion of those who were attacked. Dr. Granville proposes the use of a stimulating liniment externally, and of "alkaline drops" internally. We regret exceedingly that this talented physician should not have publicly stated the composition of these in his work, instead of referring the purchasers to Mr. Garden, in Oxford Street. How are people in Scotland, Ireland, or country-towns of England to avail themselves of these remedies? Dr. G. cannot, we are sure, intend to keep them a secret.

Dr. Fergusson, Inspector of Hospitals, and who has written so much and so well on malarious fevers and the nature and origin of malaria itself, has published two letters in the Windsor Express, (Nov. 12th and Nov. 26th,) which are extremely important. We regret exceedingly that we cannot republish them in this article, on account of the length to which the article has already extended. His letters are full of practical facts, and his conclusions are those of a man who has seen and studied the subject on the widest scale. We shall probably make use of his letters in our next number. We can only make room for a short extract here.

"There is an old term, as old as the good old English physician, Sydenham, *constitution of the atmosphere*—and to what else than to some inscrutable condition of the element in which we live, and breathe, and have our being—in fact to an atmospheric poison, beyond our ken, can we ascribe the terrific gambols of such a destroyer. 'Tis on record, that when our armies were serving in the pestilential districts in India, hundreds, without any noticeable warning, would be taken ill in the course of a single night, and thousands in the course of a few days, in one wing of the army, while the other wing, upon different ground, and consequently under a different current of atmosphere, although in the course of the regular necessary communication between troops in the field, would remain perfectly free from the disease. It would then cease as suddenly and unaccountably as it began,—attacking, weeks after, the previously unscathed division of the army, or not attacking it at all at the time, yet returning at a distant interval, when all traces of the former epidemic had ceased, and committing the same devastation. Now, will any man, not utterly blinded by prejudice, candidly reviewing these facts, pretend to say, that this could be a personal contagion, cognizable by, and amenable to, any of the known or even supposable laws of infection—that the hundreds of the night infected one another, or that the thousands of the few days owed their disease to personal communication,—as well affect to believe that the African Simoon, which prostrates the caravan and leaves the bones of the traveller to whiten in the sandy desert, could be a visitation of imported pestilence.

That the seeds of his fury have long been sown amongst us may be proved, and will be proved ere long by reference to fatal cases of unwonted Cholera Morbus appearing occasionally during the last six months in London, Port Glasgow, Abingdon, Hull, and many other places, which as it did not spread, have been passed unheeded by our health conservators; but, had the poison then been sufficiently matured to give it epidemic current, would have been blazed forth as imported pestilence. Some one or other of the ships constantly arriving from the north of Europe could easily have been fixed upon as acting the part of Pandora's box, and smugglers from her, dispatched instanter, to carry the disease into the inland quarters of the kingdom. I write in this manner, not from petulance, but from the analogy of the yellow fever, where this very game, I am now describing has so often been played with success in the south of Europe; and will be played off again, so long as lucrative boards of health and gainful quarantine establishments, with extensive influence and patronage, shall continue to be resorted to for protection against a non-existent—an impossible contagion."

We ought not to pass over an ingenious Essay by Dr. Forster, who has written largely on Atmospheric Diseases, and has recently published a pamphlet on the Epidemic Cholera. We can only briefly allude to this brochure. Dr. F. maintains that epidemics are the offspring of "an unhealthy state of the prevailing air"—that, during this state of the atmosphere, certain tribes of reptiles and insects overspread and desolate large tracts of countries—that terrestrial commotions, evinced by volcanos, &c. are now taking place in an unusual degree—that the epidemics resulting from, or accompanying these terrestial commotions, are wholly inexplicable—that they pursue a course wholly incapable of being arrested by any sanitary regulations—but that, as large cities are more frequently attacked than small country places, so the *infectious* power of the air, being there augmented by the exhalations from the bodies of patients, those who come into close proximity or contact with them, are most likely to take the disorder—this circumstance giving rise to the idea of CONTAGION, while it ought to point to ventilation and cleanliness. Dr. F.'s pamphlet, like all his other writings, evinces great research. He has been unfairly attacked by one of his townsmen.

Mr. Pettigrew, a talented surgeon, has written a popular pamphlet on the subject of cholera, which is well adapted to the object which the author had in view. His descriptions are clear—his means of cure judicious. He is evidently a non-contagionist.

Many clever papers have appeared in the public journals from anonymous correspondents—the best, perhaps, of these bears the signatures of Alpha, and also of Omega. They both carry intrinsic evidence of being the offspring of the same individual. The article Cholera in the Westminster Review is, we think, the fairest and most impartial of all those which have emanated from the advocates of contagion. Dr. Copland's article in the Foreign Quarterly is erudite and clever, but the ardour with which he advocates the cause of contagion, and the peculiarity of some of his views, detract something from the merit of his compilation.

Mr. Bell's letter to Sir Henry Halford is one of the most forcible and argumentative pamphlets which have appeared on the subject. Its chief object, however, being to annul the sanitary code issued by the first Board of Health, and that object being attained by the issue of a different code, a great portion of the pamphlet is now become comparatively uninteresting. Nevertheless we shall close this long article with a few excerptæ from Mr. Bell's Letter.

"First, then, I ask, what has our European experience of Cholera taught us? The general body of Indian practitioners had long been accustomed to regard the disease as non-contagious. But as it advanced into Europe, the eminent physicians in the several countries which it has successively ravaged, disregarded the Indian experience; and, perhaps wisely, resolved to protect themselves against its approaches by rigorous quarantine regulations, or by sanitary cordons, enforced with all the power of despotic governments. Look, however, at the result:—In the face of all such regulations the disease has advanced westward with undeviating and irresistible strides; and, so far from the experience of Europe refuting the Indian conclusions on the question of contagion, it would appear, that, as each country becomes acquainted with the disease, the conviction becomes general, that to whatever cause its dissemination is to be ascribed, it is not propagated by contagion, and cannot be confined within any prescribed limits."

"The experience of India, of Russia, and more lately of Germany, proves that Cholera travels not with the erratic course of a contagious distemper, but with a march steadily progressive in a particular direction. The absence of quarantine in India did not accelerate its progress; the enforcement of such regulations in Europe has *in no instance* retarded it. Nay more, it is well known that new cases of the disease do not occur in vessels after they have got fairly free of the port where it prevails : and in the thousands of vessels which have performed quarantine on our shores, no well authenticated case of the disease has been reported."

"A Cholera patient requires not only the almost constant attendance of a medical practitioner, but his life, in most cases, depends on the unremitted efforts of non-medical assistants. In India, we see the patient's ordinary acquaintance, free from all alarm, actively engaged in shampooing or rubbing spasmodically affected limbs ; while the medical attendant in that country has always the aid of any number of volunteers he may require. But under the influence of your regulations, where are we to look for such coadjutors ? It is impossible for an unprofessional person to read the directions lately published in the Gazette, without being impressed with the belief, that, if he touch a patient labouring under Cholera, he does little less than inoculate himself with a mortal poison. The Board themselves, indeed, are already, I much fear, paving the way for inefficiency in hospital establishments; for, by one of the published heads of instruction, the hospital establishments are directed to be *kept low*, that the number of attendants may not tend to spread the disease."

"In India, generally speaking, one in eight of the persons attacked dies : in Europe, with all the advantages of superior medical skill, the deaths, if newspaper reports are to be credited, have been nearly in the proportion of one to two; and my persuasion is, that this extraordinary mortality is to be attributed, in no inconsiderable degree, to rigorous sanitary regulations; which, while they have in no respect arrested the progress of the disease, have increased its evils, both by their direct operation and by the causeless and enervating panic which they have been so powerful a means of promoting." 7.

In an appendix to this article, we shall make some observations on the cholera as it has appeared in England ; and hope, on the whole, that our analyses and extracts, in this Number, will be found to embody a valuable mass of facts and observations on the important subject under consideration.

*(To be continued in the Periscope.)*

